# Receptor-Like Kinase RUPO Interacts with Potassium Transporters to Regulate Pollen Tube Growth and Integrity in Rice

**DOI:** 10.1371/journal.pgen.1006085

**Published:** 2016-07-22

**Authors:** Lingtong Liu, Canhui Zheng, Baijan Kuang, Liqin Wei, Longfeng Yan, Tai Wang

**Affiliations:** 1 Key Laboratory of Plant Molecular Physiology, Institute of Botany, Chinese Academy of Sciences, Beijing, China; 2 University of Chinese Academy of Sciences, Beijing, China; National University of Singapore and Temasek Life Sciences Laboratory, SINGAPORE

## Abstract

During sexual reproduction of flowering plants, the pollen tube grows fast and over a long distance within the pistil to deliver two sperms for double fertilization. Growing plant cells need to communicate constantly with external stimuli as well as monitor changes in surface tension of the cell wall and plasma membrane to coordinate these signals and internal growth machinery; however, the underlying mechanisms remain largely unknown. Here we show that the rice member of plant-specific receptor-like kinase CrRLK1Ls subfamily, *Ru**ptured* *Po**llen tube* (*RUPO*), is specifically expressed in rice pollen. RUPO localizes to the apical plasma membrane and vesicle of pollen tubes and is required for male gamete transmission. K^+^ levels were greater in pollen of homozygous CRISPR-knockout lines than wild-type plants, and pollen tubes burst shortly after germination. We reveal the interaction of RUPO with high-affinity potassium transporters. Phosphorylation of RUPO established and dephosphorylation abolished the interaction. These results have revealed the receptor-like kinase as a regulator of high-affinity potassium transporters via phosphorylation-dependent interaction, and demonstrated a novel receptor-like kinase signaling pathway that mediates K^+^ homeostasis required for pollen tube growth and integrity.

## Author Summary

The pollen tube is a representative tip-growing cell, and it grows in female reproductive tissues with amazing speed to transport two sperm cells into the embryo sac for double fertilization. This feature and function require pollen tubes to communicate with female tissues and maintain their integrity before sperm discharge. Progresses in the cell wall model and osmotic pressure dynamics have contributed to our understanding of pollen tube growth and integrity maintenance, but underlying molecular mechanisms are still remain largely unknown. This work identifies a novel CrRLK1L receptor kinase RUPO from rice, which is in a different phylogenetic branch compared with the known ANX1 and ANX2. RUPO controls high-affinity potassium transporters via phosphorylation-dependent interaction. The RUPO-potassium transporter module regulates K^+^ homeostasis for pollen tube growth and integrity. Thus, this study reports a novel CrRLK1L signaling pathway that mediates K^+^ homeostasis required for pollen tube growth and integrity. These findings provide the first evidence for involvement of receptor-like kinase CrRLK1L in K^+^ homeostasis via regulating potassium transporters.

## Introduction

A major functional innovation of spermatophytes is the evolution of pollen grains consisting of the large vegetative cell (VC) and the immobile male gametes (sperm cells). Pollen grains are tolerant of desiccation and can spread over long distances by wind and/or animal pollinators, thus being an important driving force for species diffusion. During pollination and fertilization of flowering plants, pollen grains undergo adhesion and hydration on the female stigma, then the VC bulges through the germination aperture to generate a tip-growing pollen tube (PT)[1]. The PT enters stigmatic cells, grows fast and over a long distance within the pistil via turgor-driving growth at the tip, and finally reaches the receptive synergid cell, where the tube arrests its growth and ruptures at its tip to release two sperm cells for double fertilization [2–4]. Thus, successful fertilization requires maintenance of PT integrity and timely growth arrest and rupture.

Growing plant cells need communicate constantly with external stimuli as well as monitor changes in surface tension of the cell wall and plasma membrane to coordinate these signals and internal growth machinery [5–7]. The fast tip-growing features of PTs suggest that they have active mechanisms underlying external signal sensing, coordination and response, but these mechanisms are largely unknown. In Arabidopsis, FER, ANX1/2, THE1 and HERK1/2, members of the plant-specific receptor-like kinase (RLK) of *Catharanthus roseus* RLK1-like (CrRLK1L) subfamily are implicated in cell expansion [8]. *FER* is expressed in synergids and various vegetative tissues except pollen. *FER* is required for timely growth arrest of PTs in the receptive synergid and for root hair elongation [9–12]. Conversely, *ANX1* and *ANX2* are expressed preferentially in pollen and function redundantly [13, 14]. PTs in *anx1anx2* double mutants showed precocious rupture. Overexpression of *ANXs* caused PT growth inhibition [15]. These CrRLK1L members appear to share downstream signaling components, reactive oxygen species (ROS)-producing NADPH oxidases [15]. Recently, MRI, a receptor-like cytoplasmic kinase, was identified as a positive CrRLK1L signaling component and functioned downstream of ANXs and NADPH oxidases in PTs and downstream of FER in root hairs [11]. Therefore, these CrRLK1L members regulate cell integrity possibly via the NADPH oxidase-ROS system, but their roles in PT burst remain elusive. Disruption of ANXs or NADPH oxidases caused premature burst of PTs, which suggests that ROS inhibits PT overgrowth and prevents tube rupture [15, 16]. However, studies of FER showed that promoting ROS production induced PT rupture, and scavenging of H_2_O_2_ and ·OH prevented PT bursting [17]. Thus, further insights into CrRLK1L signaling are needed to understand the biological functions of these receptors as well as the mechanisms underlying the maintenance of PT integrity, timely growth arrest and rupture.

K^+^ is an essential mineral for pollen germination and tube growth [18, 19]. Cytosolic K^+^, together with sugar, contributes to the turgor pressure of growing PTs [20–22]. Disruption of SPIK, an inward K^+^ channel in Arabidopsis, strongly reduced K^+^ influx, which resulted in impaired pollen germination and PT growth [23]. PTs lacking K^+^ transporters CHX21 and CHX23 grew down in the transmitting tract and failed to turn to the ovule [24]. Excessive inflow of K^+^ through K^+^ channel KZM1 ruptured the PT in maize [25]; blocking the opening of an outward K^+^ channel in the PM of *Pyrus pyrifolia* also resulted in PT burst [26]. These results suggest that fine-tuned K^+^ homeostasis may be important for PT growth and timely rupture.

In this study, we investigated the function of *Ru**ptured* *Po**llen tube* (*RUPO*), a novel member of the CrRLK1L subfamily from rice. *RUPO* is specifically expressed in mature and germinated pollen. The T-DNA insertional mutant *rupo+/-* is defective in *rupo* male gametophyte transmission. Loss-of-function mutants generated with the CRISPR method showed significantly greater K^+^ content in mature pollen than wild-type plants and precocious PT rupture shortly after germination. This protein is localized in the PM and vesicle of PTs and interacts with K^+^ transporters OsHAK1, OsHAK19 and OsHAK20, which indicates that the CrRLK1L subfamily regulates K^+^ transporters to control PT integrity in rice.

## Results

### RUPO is required for pollen function and expressed specifically in pollen

**Fig 1:**
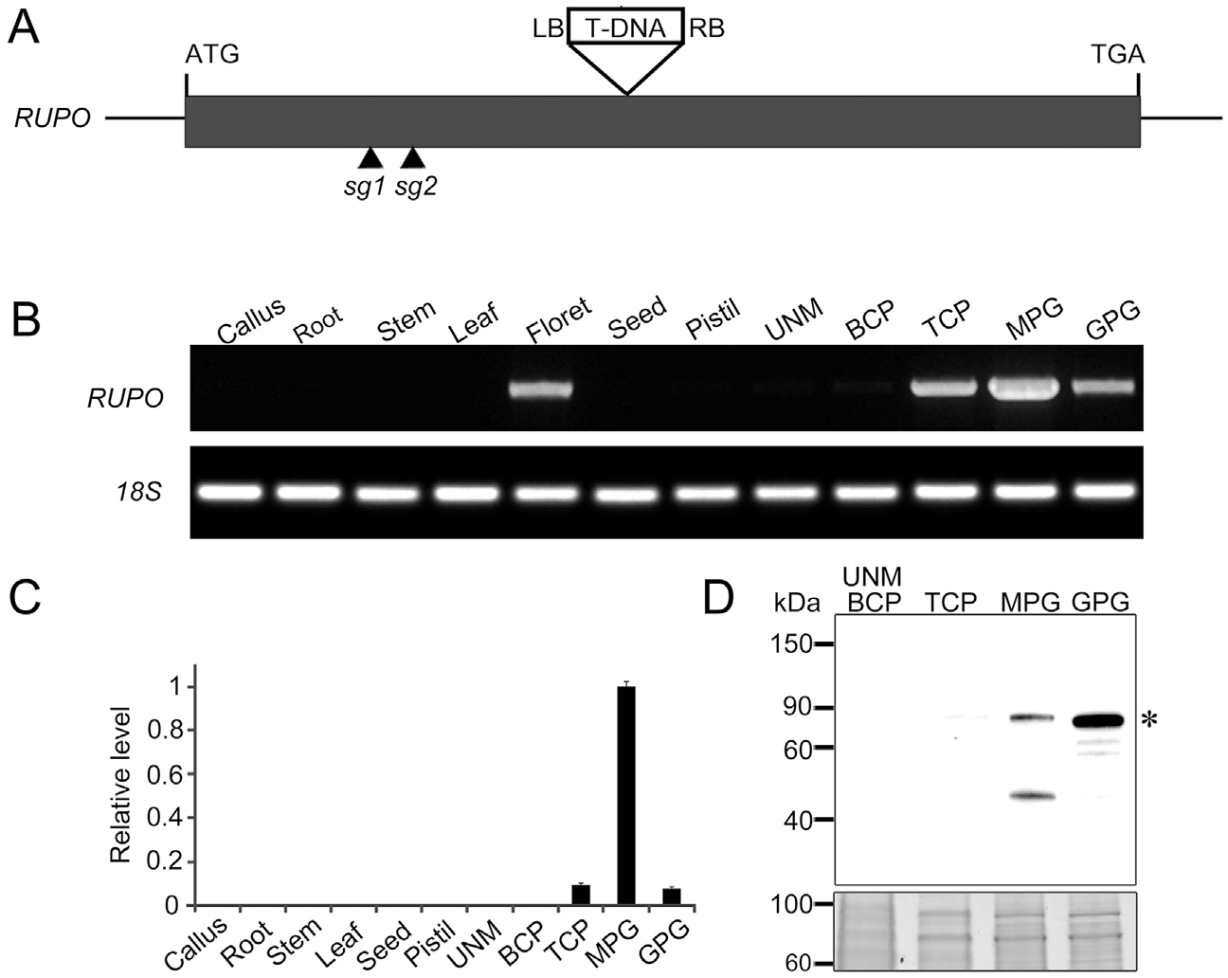
RUPO is expressed specifically in rice pollen. (A) The genomic structure of *RUPO* and positions of T-DNA insertion site and CRISPR target sites (*sg1* and *sg2*). *RUPO* is an intronless gene. The black rectangle represents the exon. (B) RT-PCR and (C) quantitative PCR analysis of RUPO transcripts in different tissues. 18S ribosomal RNA was used as an internal control. The relative expression is presented as mean ± s.e.. (D) Western blot analysis of RUPO protein in pollen. 20 μg of total proteins from pollen was loaded in each lane, resolved on 4~15% SDS-PAGE gel and detected with anti-RUPO polyclonal antibody. Asterisk indicates RUPO band. Upper panel: western blot; bottom panel: protein loading control stained with Coomassie blue. UNM, uninucleate microspore; BCP, bicellular pollen; TCP, tricellular pollen; MPG, mature pollen grain; GPG, germinated pollen grain.

In previous study of transcriptomic profiles of developing and germinated rice pollen[27], we revealed a rice CrRLK1L member[28] with specific expression in mature and germinated pollen and obtained a heterozygous T-DNA insertional mutant, *rupo+/-* (details follow) from the POSTECH database[29,30]. The heterozygous mutant contains a single T-DNA insertion in the exon region (Fig 1A) and does not substantially differ from the wild type (WT) in vegetative and reproductive growth (S1 Fig). However, on self-fertilization, the progeny displayed a distorted segregation ratio for the *rupo+/-* mutant to the WT of 1:1.07 (no homozygous mutants), rather than the expected 1:2:1 Mendelian ratio (Table 1), which indicates a gametophytic defect. To investigate whether the gametophytic defect was in the male or female, we performed reciprocal crosses of *rupo+/-* with WT plants. When *rupo+/-* pistils were pollinated with WT pollen, the progeny produced *rupo+/-* and WT plants at a ratio of 1:1.12. However, when WT pistils were pollinated with pollen from *rupo+/-*, all progeny were WT plants (Table 1). These results indicate defective male gametophyte transmission.

**Table 1:** Segregation analysis of heterozygous *rupo*+/- mutant.

Genotype of parents		Genotype of progeny			Observed ratio	Expected ratio
♀	♂	WT	*+/-*	*-/-*	WT: *+ / - *: -/-	WT: *+ / - *: -/-
*RUPO / rupo*	*RUPO / rupo*	301	322	0	1:1.07:0^a^	1:2:1
*RUPO / rupo*	*RUPO/RUPO*	180	202	0	1:1.12:0^b^	1:1:0
*RUPO / RUPO*	*RUPO / rupo*	214	0	0	214:0:0 ^a^	1:1:0
Complementation	*RUPO/rupo +gRUPO* × self	11	7	5	11:7:5^b^	2:3:1

^a^Significantly different from the Mendelian segregation ratio (χ^2^, P<0.01)

^b^Not significantly different from the Mendelian segregation ratio (x^2^, P > 0.05).

To confirm that the defective male transmission was caused by dysfunction of the *RUPO* gene, we introduced a *RUPO*::RUPO transgene into *rupo+/-* plants (S1 Fig). Because *rupo+/-* already contains hygromycin resistance conferred by the original T-DNA insertion, G418 (kanamycin) resistance was used in screening transgenic plants (T0 generation). T1 plants carrying both the hygromycin and G418 resistance were identified by PCR analysis. In the T1 generation, 5 homozygous *rupo-/-* in 23 plants were obtained (Table 1). Therefore, WT *RUPO* was able to rescue the defect in the *rupo* male transmission.

RUPO transcripts were not detected in pistils and vegetative tissues but highly expressed in florets, tricellular pollen (TCP), mature pollen grains (MPGs) and germinated pollen grains (GPGs), with the strongest signals in MPGs (Fig 1B and 1C), so *RUPO* was specifically expressed in pollen. Consistently, transgenic rice lines harboring *RUPO*::GUS showed strong GUS signals in pollen grains but undetectable signals in pistils (S2 Fig). Furthermore, we developed a polyclonal antibody against RUPO (S3 and S4 Figs). Immunoblot assay detected RUPO protein in MPGs, with high enrichment in GPGs, but barely detected in immature pollen (Fig 1D). This pollen-specific expression of *RUPO* is consistent with its requirement for male transmission.

### *rupo* PTs rupture *in vitro*

**Fig 2:**
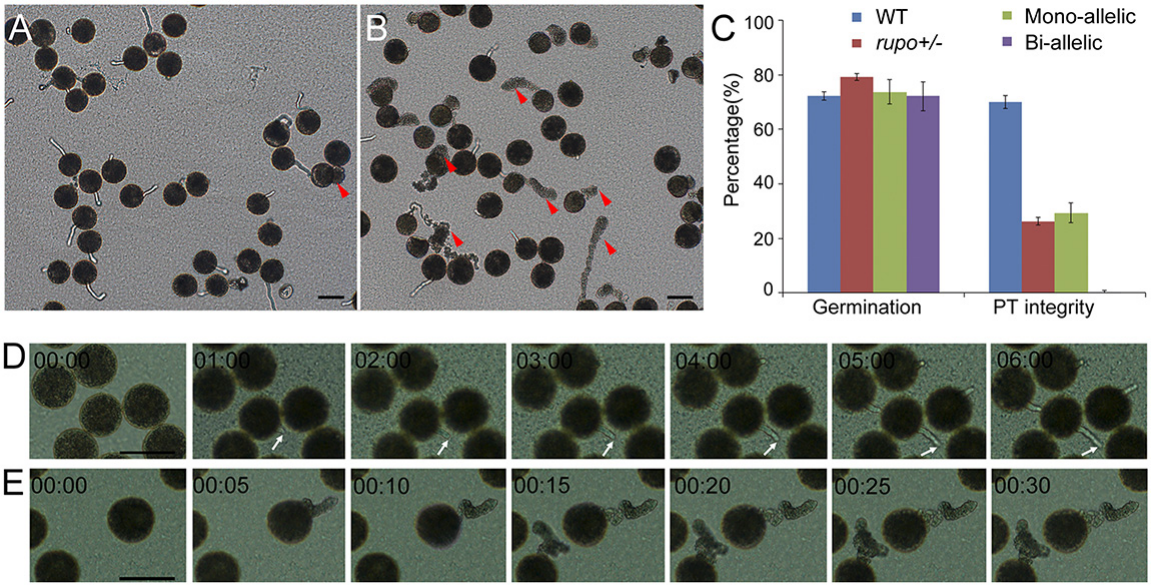
In vitro pollen germination and growth assay. (A) Wild-type pollen. (B) *rupo+/-* pollen. Red arrowheads indicate ruptured pollen tubes. (C) Pollen germination rate and percentage of pollen tube integrity. The results are presented as mean±s.e. (D) Time-lapse images of wild-type pollen germinating on agarose medium. White arrows indicate the growth of a wild-type pollen tube. (E) Time-lapse images of bi-allelic pollen germinating on agarose medium. Note that *rupo* pollen tubes burst and released cell contents in seconds without forming any visible pollen tubes.Time is indicated in minutes:seconds format. Scale bars, 50 μm. (see also S1 and S2 Movies in the supplementary material).

To investigate how *RUPO* affects male transmission, we first examined the morphological features of MPGs. Pollen grains from both *rupo+/-* and WT plants contained one loosely stained vegetative nucleus and two condensed sperm nuclei and were viable (S5 Fig). The pollen coat, wall, and germination aperture did not differ between *rupo+/-* and WT plants (S5E–5J Fig). Therefore, *rupo* pollen grains develop normally and are viable. Next, we examined in vitro pollen germination and tube growth on solid germination medium. In this condition, 72.3% WT pollen germinated, and 70% germinated WT pollen had integral PTs (Fig 2). In contrast, approximately 79.2% *rupo+/-* pollen germinated, comparable to that of WT pollen, but only 26.3% germinated *rupo+/-* pollen had integral PTs (Fig 2). The *rupo* mutation may not affect pollen germination but impairs the integrity of newly generated PTs.

To study *rupo* phenotypes in detail, we used a CRISPR-Cas9 system [31, 32] to generate two independent homozygous mutant lines, *rupo-sg1 and rupo-sg2* (S6 and S7 Figs), that harbor frame-shift mutations (most were one-base-pair insertion or deletion) at respective target sites *sg1* (532–551 bp) and *sg2* (603–622 bp) in *RUPO* (S6 Fig). We obtained 10 independent homozygous bi-allelic plants (*rupo-/-)*, 6 heterozygous mono-allelic plants (*rupo-/+)* and 5 chimerical plants (excluded from further analysis). We examined the in vitro germination behavior of bi-allelic and mono-allelic pollen. Bi-allelic and mono-allelic plants had similar pollen germination rate, comparable to WT plants. Most WT PTs maintained integrity (S8A Fig), even after germination for 40 min (PTs of rice usually stop growing after germination for 15~20 min in vitro). In contrast, all of the bi-allelic pollen burst within the first 5 min after germination (S8C Fig, S1 and S2 Movies). As expected, approximately 29.3% of mono-allelic pollen had integral PTs (S8B and S8D Fig), which was similar to the T-DNA insertion mutant *rupo+/-* (26.3%; Fig 2C), because mono-allelic plants should genotypically mimic the T-DNA insertion heterozygous *rupo+/-*. These data demonstrate that the loss-of-function *rupo* allele led to PT burst.

### *rupo* PTs rupture in the pistil

**Fig 3:**
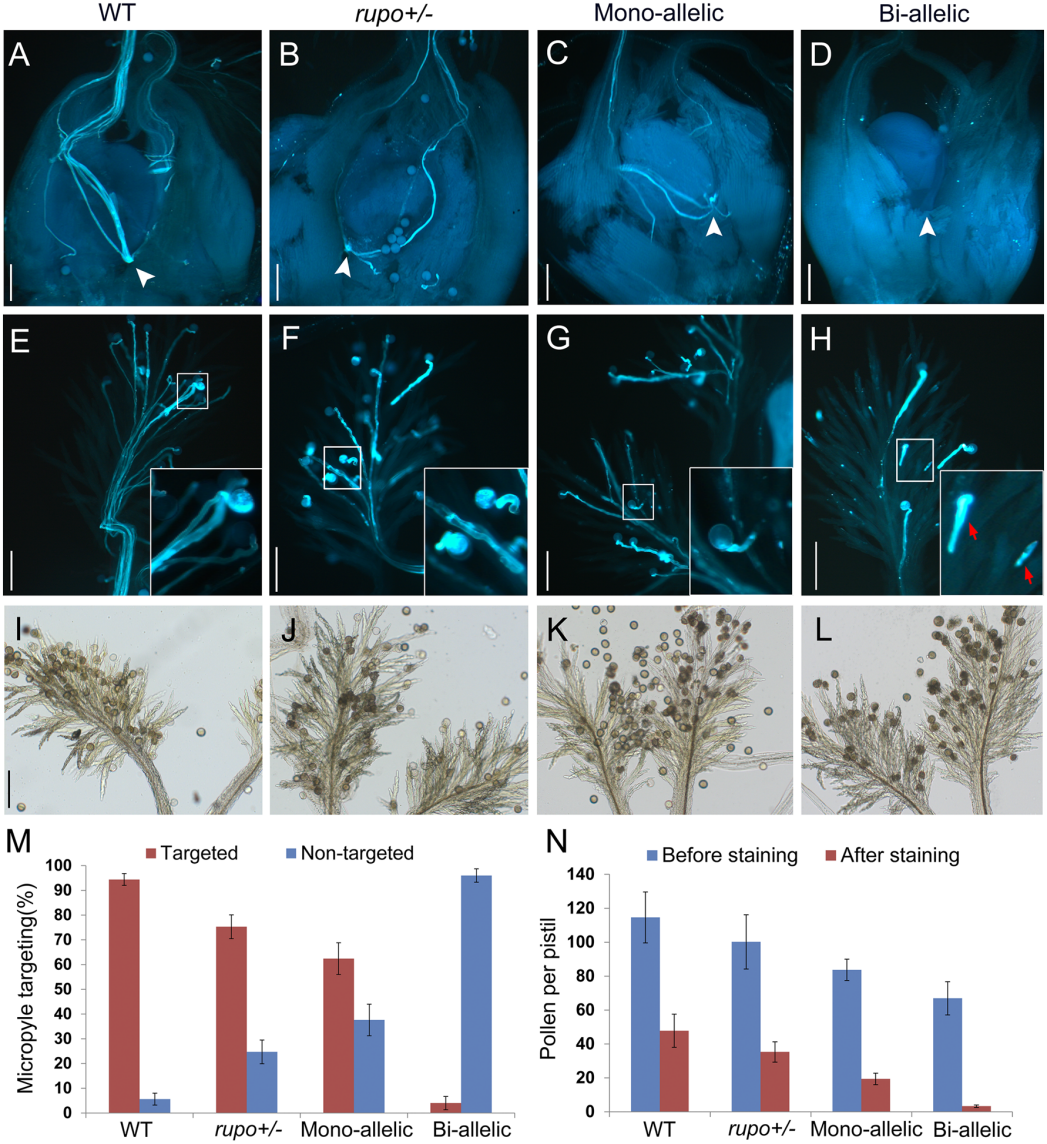
Homozygous bi-allelic mutant pollen tubes burst in pistils. (A to H) Pollen tube growth in pistils of wild-type (A,E), heterozygous T-DNA mutant *rupo+/-* (B,F), mono-allelic mutant (C,G) and bi-allelic mutant (D,H). White arrowheads indicate the putative positions of micropyles. All ovules were targeted by pollen tubes except for ovules from the bi-allelic mutant. Inserts in (E,F,G,H) are close-ups of representative pollen tubes in wild-type (E), *rupo+/-* (F), mono-allelic (G) and bi-allelic (H) stigmas. Red arrows in (H) indicate callus spots. (I,J,K,L) Bright-field images of representative pistils without aniline blue staining from wild-type (I), *rupo+/-* (J), mono-allelic (K) and bi-allelic (L) plants. Scale bars, 100 μm. (M) The percentage of ovules targeted by pollen tubes. n = 109 for wild-type pistils, n = 106 for *rupo+/-* pistils, n = 95 for mono-allelic pistils and n = 163 for bi-allelic pistils. (N) Pollen number per pistil before or after aniline blue staining. n = 79 (before staining) or 75 (after staining) for wild-type pistils, n = 74 or 107 for *rupo+/-* pistils, n = 59 or 89 for mono-allelic pistils, and n = 80 or 145 for bi-allelic pistils. Results are presented as mean ± s.e..

We used self-pollinated pistils from WT, *rupo+/-*, and bi-allelic and mono-allelic plants to examine the in vivo tube growth behavior by aniline blue staining. In WT pistils, PTs penetrated the stigma and targeted the ovule. In WT plants, 94.4% of ovules (n = 109) were targeted by at least one PT (Fig 3A and 3M). Surprisingly, in bi-allelic plants, only 4% of ovules (n = 163) were targeted by PTs (Fig 3D and 3M). On closer examination, 73.3% bi-allelic pistils (n = 163) were attached with callus spots instead of PTs and pollen grains, and the remaining 22.7% pistils (n = 163) contained PTs that prematurely stopped in papilla cells or transmitting tracts (Fig 3H), so the PT ruptured in pistils. We also observed the pollen amount on pistils. After aniline blue staining, a WT pistil contained 47.8 pollen grains, on average (Fig 3E and 3N), but a bi-allelic pistil had only 3.3 pollen grains, on average (Fig 3H and 3N).

To clarify whether aniline blue staining affected number of pollen grains in bi-allelic pistils, we collected self-pollinated pistils and counted pollen grains attached to the pistils directly or after aniline blue staining (Fig 3I–3L). On average, 47.8 and 114.6 pollen grains were observed in treated and untreated pistils, respectively, of WT plants, whereas the respective pollen amount was decreased in *rupo*+/- and mono-allelic plants and significantly decreased in bi-allelic plants, with 3.3 and 66.9 pollen, on average, in treated and untreated pistils, respectively (Fig 3N), which suggests sufficient pollen adhering to bi-allelic pistils. In addition, Alexander staining showed that the pollen amount in bi-allelic anthers before or after dehiscence was similar to that of the WT (S7 Fig). These data indicate that *rupo* pollen tubes burst in vivo and poorly adhered to pistils after staining. Adherence defect of *rupo* pollen is probably caused by the failure of tube invasion of papillae cells, for ruptured pollen is easier to wash out (S9 Fig). Furthermore, we performed reciprocal crossing between WT and bi-allelic plants to ensure the PT burst was not induced by abnormal pistils of bi-allelic plants. When bi-allelic pistils were pollinated with WT pollen, they produced normal seeds. However, when WT pistils were pollinated with bi-allelic pollen, no seeds were produced (S10 Fig). Therefore, *rupo* PTs burst *in vivo*.

### RUPO is localized to the PT tip

**Fig 4:**
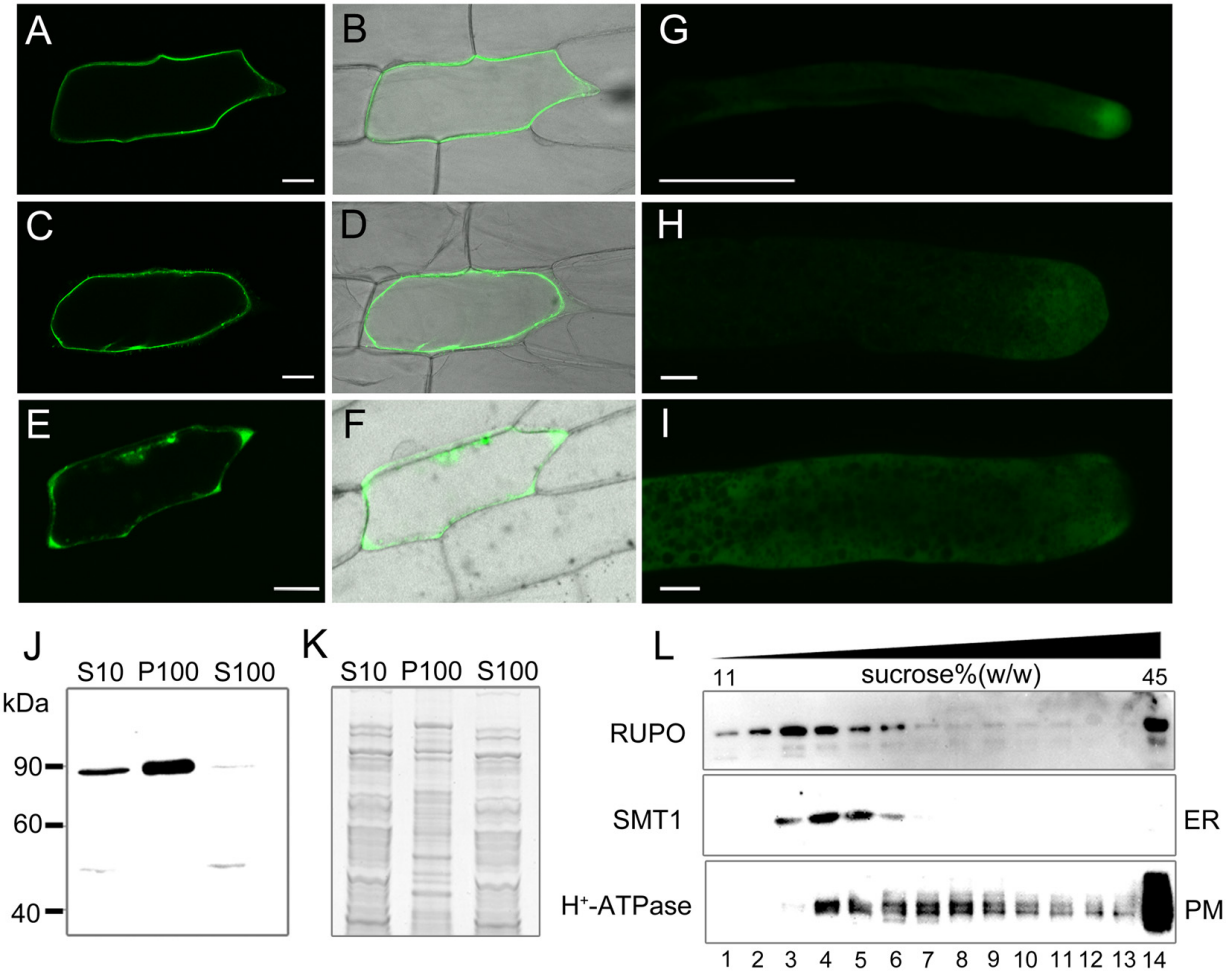
RUPO localizes to the plasma membrane and cytoplasmic vesicles of the pollen tube. (A-F) Subcellular localization of the *35S*::RUPOΔC-GFP fusion protein in onion epidermal cells. (A) Single confocal section and (B) bright-field image of the cell bombarded with *35S*::RUPOΔC-GFP plasmid. (C,D) The same cell as in (A,B) was treated with 0.8 M mannitol to induce plasmolysis. (E) Single confocal section and (F) bright-field image of the cell bombarded with *35S*::GFP. (G,H,I) Subcellular localization of the *Ubi*::RUPO-GFP fusion protein in lily pollen tubes. (G,H) *RUPO* or (I) *GFP* alone driven by ubiquitin promoter was transiently expressed in lily pollen tubes. Scale bars, 50 μm in (A) to (G), 5 μm in (H,I). (J-K) RUPO was enriched in membrane fraction. Homogenate from mature pollen grains was centrifuged at 10,000×g, and resultant supernatant (S10) was further centrifuged at 100,000×g to separate into supernatant (S100) and membrane fraction (P100). Proteins in each fraction were resolved by SDS-PAGE and probed with anti-RUPO antibody. 30 μg soluble protein (S10, S100) and 10 μg membrane protein (P100) were loaded. (J) Western blot image, (K) Protein loading control image stained with Coomassie brilliant blue. (L) Crude membrane vesicles were fractionized on a discontinuous sucrose density gradient, and proteins in individual fractions were probed with antibodies against RUPO, ER marker protein SMT1 and PM marker protein H^+^-ATPase.

We first bombarded onion epidermal cells with *35S*::RUPOΔC-GFP(the kinase domain replaced by a GFP) and with the control *35S*::GFP. Plasmolysis treatment showed that RUPOΔC-GFP was localized to the plasma membrane (PM) (Fig 4A–4D), whereas the control GFP was observed in the cell periphery and cytoplasm (Fig 4E and 4F). Next, we transiently introduced *Ubi*::RUPO-GFP and *Ubi*::GFP to lily pollen tubes. RUPO-GFP was observed at the growing PT tip (Fig 4G and 4H). By contrast, control GFP was detected throughout the PT (Fig 4I). Moreover, closer examination of the tip and shank region using variable angle TIRF microscopy showed that RUPO-GFP signals existed not only in the PM, but also in vesicles (S3 and S4 Movies). Finally, we fractionated membrane vesicles from MPGs by discontinuous sucrose density gradient. Immunoblot assay showed that RUPO was enriched in the PM, the endoplasmic reticulum and vesicles with low buoyant density (Fig 4J–4L). In conclusion, RUPO protein is a PM-localized protein and shows apical localization in PTs.

### RUPO interacts with potassium transporters and regulates K^+^ homeostasis

**Fig 5:**
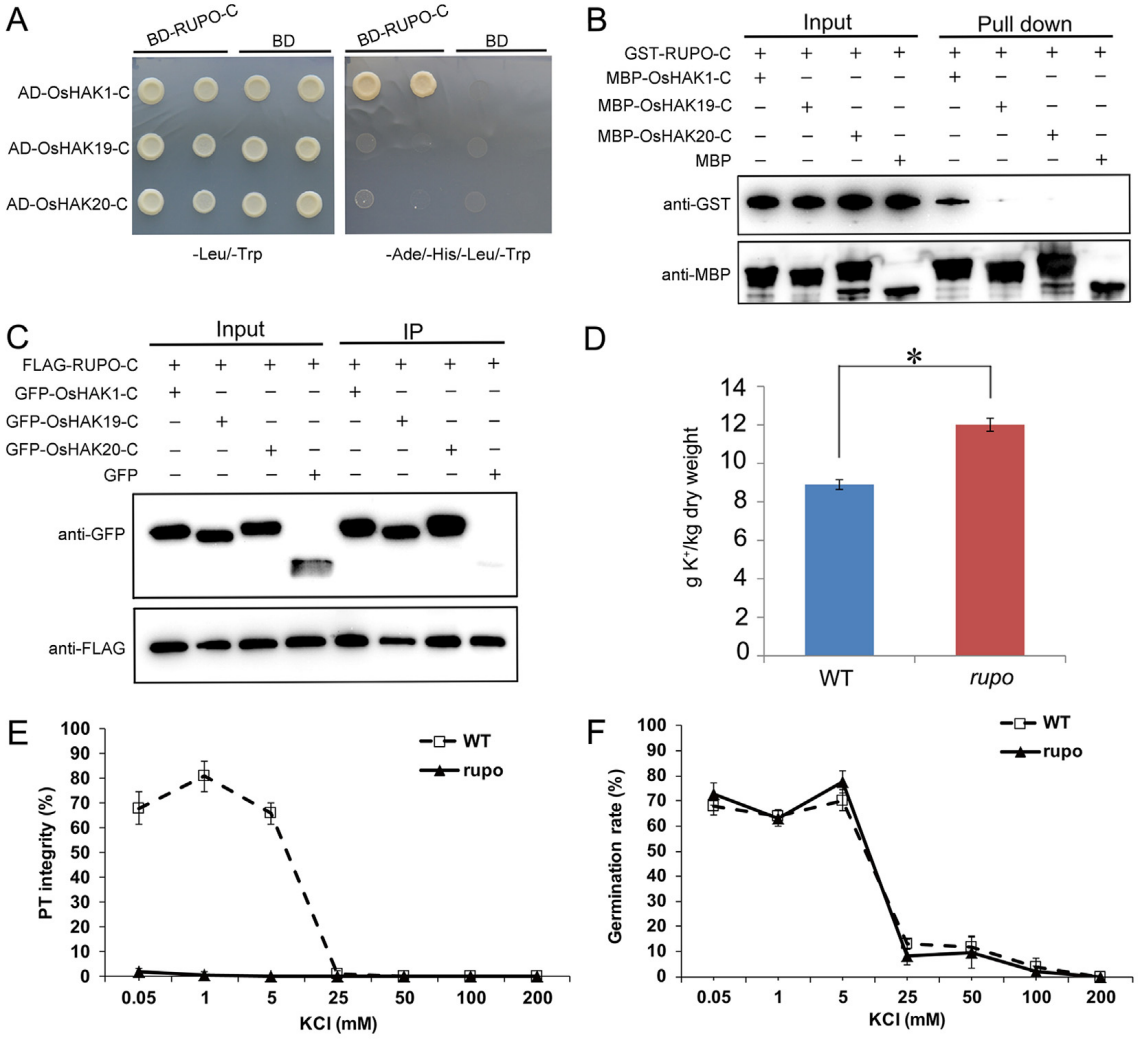
RUPO interacts with potassium transporters. (A) The intracellular domain (without juxtamembrane region) of RUPO interacts with OsHAK1-C in yeast two-hybrid assay. (B) In vitro pull-down assay of RUPO and OsHAK1/19/20 interaction. GST-RUPO-C or GST was detected by anti-GST antibody. MBP-OsHAK-C or MBP was detected by anti-MBP antibody. (C) Co-immunoprecipitation assay of RUPO and OsHAK1/19/20 interaction. (D) Determination of K^+^ in MPGs from wild-type and bi-allelic plants by inductively coupled plasma atomic emission spectroscopy (ICP-OES). Results are presented as mean ± s.e.. Student’s test *p<0.05. (E) Effect of K^+^ on pollen tube integrity in vitro. (F) Effect of K^+^ on pollen germination in vitro. Each data point is the result of at least four replicates.

To understand how *RUPO* is responsible for PT integrity, we identified potential RUPO-interacting proteins by use of the prey cDNA library prepared with mRNAs from GPGs and the bait containing the intracellular domain (without juxtamembrane region, JM) (BD-RUPO-C). We frequently identified (16 of 96 in-frame clones) cDNA insertions corresponding to the C-terminus of high-affinity K^+^ transporter OsHAK1, with the longest cDNA insertion encoding the 110-aa residue C-terminal tail of OsHAK1. OsHAK1 and its two closest homologues, OsHAK19 and OsHAK20, in rice share 77% and 75% amino acid sequence identity, respectively, and have multiple transmembrane regions (S11 and S12 Figs). Therefore, we tested RUPO-C interaction with the 110-aa C-terminus of OsHAK1/19/20 (OsHAK1-C, OsHAK19-C and OsHAK20-C). Yeast two-hybrid assay showed that RUPO-C interacted with OsHAK1-C but not OsHAK19-C and OsHAK20-C (Fig 5A). Consistently, pull-down assay showed that GST-RUPO-C specifically interacted with MBP-OsHAK1-C but not MBP-OsHAK19-C or MBP-OsHAK20-C (Fig 5B). However, when FLAG-RUPO-C was transiently co-expressed with GFP-OsHAK1-C, GFP-OsHAK19-C or GFP-OsHAK20-C in rice protoplasts, FLAG-RUPO-C interacted with GFP-OsHAK1-C and also GFP-OsHAK19-C and GFP-OsHAK20-C (Fig 5C). Therefore, RUPO physically associated with all the three K^+^ transporters, with strong interaction with OsHAK1.

In rice, *OsHAK1/19/20* belong to a KT/HAK/KUP family of 27 members which is classified into 4 clusters (I–IV) [33, 34]. Because OsHAK1/19/20 all come from the same cluster I, we further cloned another 5 OsHAKs (OsHAK26, Cluster I; OsHAK23, Cluster II; OsHAK17, Cluster III; OsHAK6, OsHAK25, Cluster IV) that expressed in rice pollen, and test whether RUPO-OsHAK interaction is general to all OsHAK members or specific to OsHAK1/19/20 with Yeast two-hybrid. When the C-tail of OsHAKs was truncated to a minimal 77 aa, OsHAK1/19/20 interacted with RUPO-C (S13A Fig). However, only the 110-aa C-tail of OsHAK1 specifically interacted with RUPO-C in yeast (S13B Fig), consistent with above results, but those of other OsHAKs did not interact with RUPO-C (S13A and S13B Fig). Together, these results suggest interaction of RUPO with OsHAK1/19/20 is specific.

Cluster I members of the KT/HAK/KUP family are involved in high-affinity K^+^ uptake, and function at low external K^+^ concentration and transport K^+^ into the cytosol against an electrochemical gradient [35, 36]. OsHAK1-GFP displayed PM localization in onion epidermal cell (S14 Fig), which is consistent with the result described by Chen et al. [37]. Similarly, OsHAK19-GFP was enriched in the PM. While OsHAK20-GFP signal was most prominent in the cytoplasm. In lily pollen tubes, OsHAK1/19/20-GFP were observed both in the cell periphery and cytoplasm (S14 Fig). K^+^ transport activity assay showed that OsHAK1 could complement the yeast mutant R5421 which lacks PM-localized K^+^ transporter TRK1 & TRK2 and requires high extracelluar K^+^ (>10 mM) for normal growth[37,38]. OsHAK19 showed a weaker complementation, and R5421 transformed with OsHAK20 could not grow under low K^+^ condition(<5 mM) (S15 Fig).

To test whether K^+^ uptake was affected in *rupo* pollen, we used atomic emission spectroscopy to determine K^+^ content in pollen grains from WT and bi-allelic plants. The mean K^+^ content was 8896 ±182 mg/kg in WT pollen but 12005 ± 241 mg/kg in *rupo* pollen, 35% higher in *rupo* than WT pollen (Fig 5D). Thus, disruption of RUPO led to over-accumulation of K^+^ in pollen. RUPO may control K^+^ homeostasis by interacting with potassium transporters. Moreover, we investigated the effect of K^+^ on PT growth and integrity. As shown in Fig 5E, 5F and S16 Fig, PT integrity and germination rate of WT were approximately 70% under the condition of 0.05~5 mM KCl, however, an increase of K^+^ to 25 mM significantly decreased the PT integrity of WT to about 1%. Notably, the pollen germination of WT and *rupo* were also severely inhibited when the medium contained 25~200 mM K^+^ (Fig 5F and S16 Fig). These data indicate that high K^+^ results in *rupo* phenotype.

### RUPO is an active protein kinase and capable of intramolecular phosphorylation

**Fig 6:**
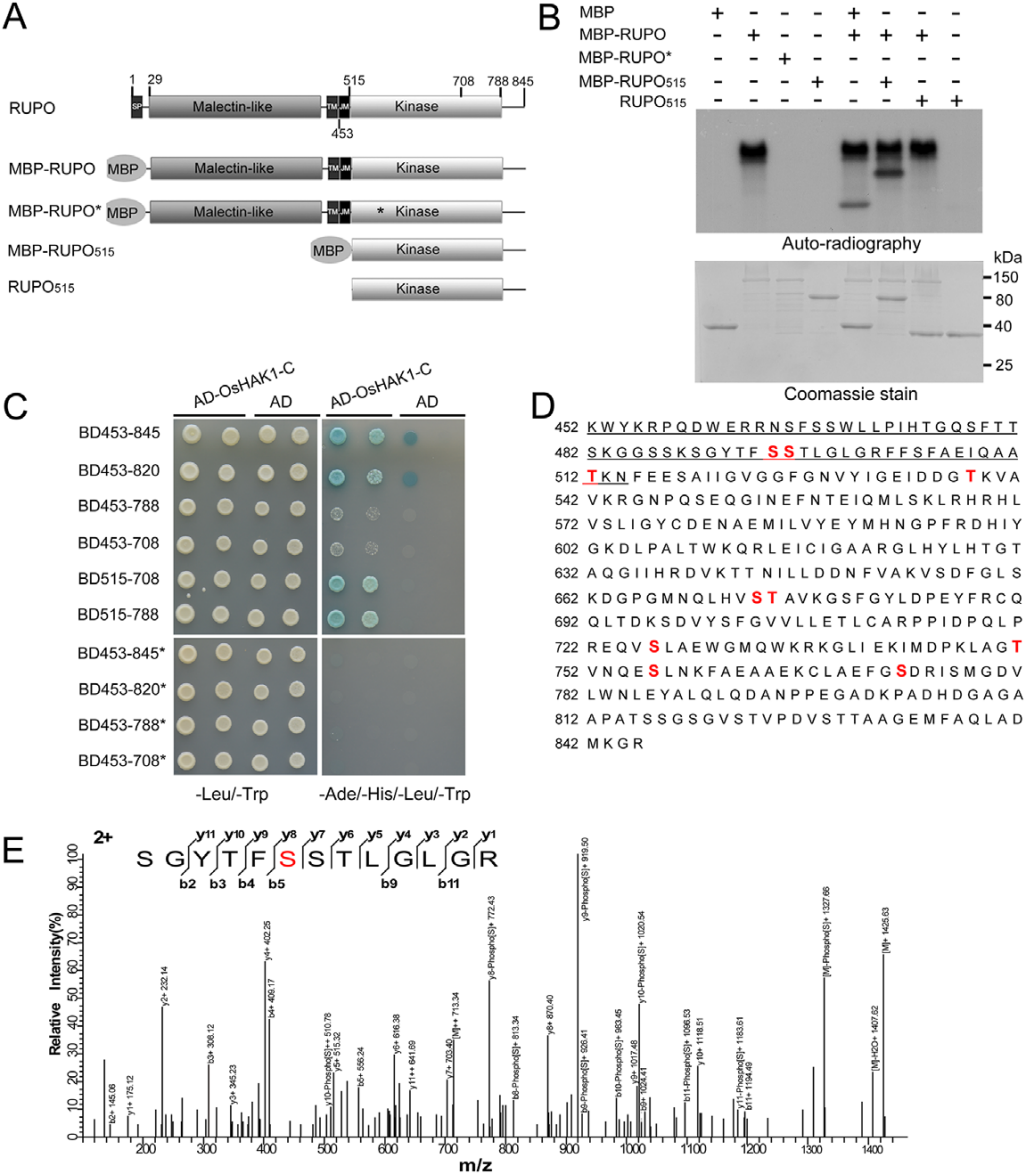
Phosphorylation regulates RUPO-OsHAK interaction. (A) Schematic diagram of RUPO used in the kinase assay and yeast-two hybrid assay. SP, signal peptide; TM, transmembrane domain; JM, juxtamembrane region. MBP, maltose binding protein. (B) The full-length RUPO (the 28 aa signal peptide is not included in this construct) is autophosphorylated, whereas its K543R point mutation version RUPO*, and JM-truncated intracellular region (MBP-RUPO_515_, and RUPO_515_) have no kinase activity. RUPO also is capable of phosphorylating MBP and MBP-RUPO_515_ but not RUPO_515_. (C) K543R point mutation abolishes the interaction of RUPO with OsHAK1 in yeast. JM and C-terminal region (788-845aa) are important elements affecting the interaction of RUPO and OsHAK1. (D) MS/MS identification of phosphorylation sites in the intracellular domain of RUPO. Phosphorylated residues are highlighted in bold red. JM region is underlined. (E) A representative MS/MS spectrum of identified phosphopeptides. The matched b and y ions are indicated.

To examine RUPO kinase activity, we expressed and purified RUPO full-length and truncated versions fused to maltose binding protein (MBP) (Fig 6A). Notably, significant cell lysis of *Escherichia coli* cells occurred within the first hour of IPTG-induced expression of MBP-RUPO, which indicates its cell toxicity. The complete intracellular domain (453~845 aa) of RUPO was lethal in *E*. *coli* cells. Therefore, we expressed a JM-truncated intracellular domain (515–845 aa) (MBP-RUPO_515_ or RUPO_515_) for phosphorylation assay.

Autoradiography showed phosphorylation of MBP-RUPO with [γ-^32^P] ATP in vitro but not its K543R version (aa substitution in ATP binding site) and the JM-truncated intracellular domain versions. The undetectable autophosphorylation of the JM-truncated intracellular domain was probably due to lack of JM, which may be necessary for autophosphorylation (Fig 6B). To elucidate whether autophosphorylation occurs in an intramolecular or intermolecular manner, we incubated MBP-RUPO with its JM-truncated intracellular domain (MBP-RUPO_515_ or RUPO_515_). MBP-RUPO could incorporate [γ-^32^P] into itself or the MBP tag but not into the separate JM-truncated intracellular domain (Fig 6B), which suggests autophosphorylation of RUPO in an intramolecular manner. The undetectable intermolecular phosphorylation in the JM-truncated intracellular domain may result from the presence of phosphorylation sites only in JM. Thus, we enriched phosphorylated RUPO peptides for MS/MS analysis. We identified 10 Ser/Thr phosphorylation sites in the intracellular domain: 3 in JM and the remaining 7 in the kinase domain and C-terminal region (Fig 6D and 6E, S1 Table). Hence, RUPO is a functional kinase capable of intramolecular phosphorylation.

Next, we determined whether autophosphorylation was necessary for the RUPO interaction with OsHAK1. We designed serial truncations of the intracellular domain of RUPO from the respective N- and C-termini (Fig 6A and 6C). The minimal kinase domain (515–708 aa, predicted by NCBI CDD; 515–788 aa, predicted by SMART and PROSITE) alone interacted with OsHAK1 in yeast. Interestingly, the presence of JM significantly reduced the interaction of RUPO (BD453-708, BD453-788) with OsHAK1, which suggests that JM has an inhibitory effect on the RUPO-OsHAK interaction (Fig 6C). However, both the intracellular domain (BD453-845) and its truncated version (BD453-820) with JM still interacted with OsHAK1. Because JM is phosphorylated during autophosphorylation of RUPO, we hypothesized that the phosphorylation on JM relieved its inhibitory effect on the RUPO-OsHAK interaction. Therefore, we examined interaction of four K543R mutants (BD453-845*, BD453-820*, BD453-788*, BD453-708*) with OsHAK1. The destruction of RUPO autophosphorylation virtually abolished all growth of yeast on quadruple dropout medium (Fig 6C). Therefore, JM plays roles in inhibiting the interaction of RUPO with OsHAK1, and phosphorylation of JM relieves the inhibition effect.

## Discussion

Studies of Arabidopsis have revealed that ANX1/2 and their closest homolog FER of the CrRLK1L subfamily play important roles in PT tip growth possibly via the downstream NADPH oxidases [10, 11, 13, 17]. Here, we identified a novel CrRLK1L member, RUPO, in the monocot rice. The RUPO signaling pathway for PT growth and integrity may differ from that of ANX1/2 and FER. First, *RUPO* is expressed specifically in pollen, whereas ANX1/2 transcripts are highly detectable in pollen and moderately detectable in vegetative tissues [13], and *FER* is expressed in various tissues except in pollen [10]. Second, *RUPO* is in a separate subclass, relative to *ANX1/2* and *FER*, of the phylogenetic tree [39]. Thus, *RUPO* is not an ortholog of *ANX1* and *ANX2* but rather is very close to At2g21480 and At4g39110 that are both expressed specifically in pollen of Arabidopsis [39, 40]. *ANX1/2* and *FER* have rice orthologs: Os05g20150 and Os03g21540/Os01g56330, respectively [39]. Third, most *rupo* pollen tubes burst immediately after germination in vitro. In vivo, most *rupo* pollen grains discharged on germination or PTs ruptured before growing into the stigma. The *rupo* phenotypes were similar to but appeared to occur earlier than those of *anx1anx2* [13, 14]. Finally, RUPO is required for PT growth and integrity via downstream component K^+^ transporters. So, monocots and eudicots may have multiple CrRLK1L signaling pathways to orchestrate PT growth and cross-talk between PTs and female cells, and these pathways may further differentiate after the monocot–dicot split, which occurred 160 million years ago[40].

Phosphorylation and dephosphorylation are important mechanisms to regulate activities of plant K^+^ channels [41, 42]. To our knowledge, no study has investigated the involvement of RLKs in regulating plant K^+^ transporters. RUPO is a functional kinase and physically interacts with potassium transporters OsHAK1, OsHAK19 and OsHAK20, with strong interaction with OsHAK1, via the kinase domain. The inconsistent results between Y2H, pull down and IP were possibly due to post-translational modifications or proper folding of OsHAK-C in different systems. The RUPO-OsHAK1 interaction depends on phosphorylation in the RUPO intracellular domain, which indicates that autophosphorylation modifies RUPO status and in turn regulates OsHAK1 activity via physical interaction. Consistent with this finding, *rupo* pollen contained 35% greater K^+^ than WT pollen, which suggests that RUPO negatively regulates OsHAK activities.

*OsHAK1/19/20* are phylogenetically placed into one clade and function in high-affinity K^+^ uptake. Available public data show that *OsHAK1* is universally expressed in vegetative and reproductive organs, with high levels in roots and anthers; *OsHAK20* shows preferential expression in anthers and stigma (http://www.bar.utoronto.ca/efprice/cgi-bin/efpWeb.cgi) and *OsHAK19* shows preferential expression in anthers (http://rice.plantbiology.msu.edu/index.shtml). We detected the full-length transcripts of all three genes in mature rice pollen. OsHAK1 and OsHAK19 appeared to localize in PM and could complement yeast mutant defective in K^+^ uptake. Knockout of OsHAK1 reduced K^+^ uptake in rice roots by ~80% under low exogenous K^+^ [37]. Thus, OsHAK1 may be an important K^+^ transporter for K^+^ homeostasis. Generally, the plant cytoplasm maintains a tightly regulated K^+^ concentration [42]. K^+^ is the major osmotically active solute to maintain plant cell turgor and drives cell expansion. K^+^ homoeostasis plays central roles in pollen hydration, germination and PT growth, and osmotic processes are important driving forces for PT growth [23, 24, 43–46]. In maize, the application of synergid-expressed ZmES4 triggered the opening of PT-expressed K^+^ channel KZM1, thus leading to rapid PT burst [25]. Our study shows the correlation of K^+^ over-accumulation and burst on germination of *rupo* pollen. Changes in K^+^ homeostasis in pollen and PTs may represent a physiological mechanism to regulate PT growth and burst by affecting osmotic pressure. Although there was only a mild increase (35%) in the overall K^+^ inside the *rupo* pollen grains, the cytoplasmic K^+^ level may further increase during the hydration and germination process. It was also possible that at the growing pollen tube tip, much higher osmotic pressure might be induced due to an uneven distribution of K^+^. Currently two different models, the cell wall model and hydrodynamic (osmotic) model are commonly used to explain the mechanism of pollen tube growth and discharge [47]. The previously well characterized CrRLK1L members ANX1/2 suggest that Arabidopsis PT burst is probably caused by cell wall damage triggered by ROS. However, our research suggests rapid osmotic pressure change may also contribute to PT rupture in monocot rice. In fact, osmotic shock is an effective method to isolate sperm cells from PTs [48–51]. In low-osmotic solution, PTs of tomato and lily burst easily by rapid water influx, emitting sperm cells and cytoplasm. These results suggest osmotic pressure alone is sufficient to induce PT burst. It will be very interesting to see whether CrRLK1Ls use both or distinct mechanisms to burst the pollen tube in a plant species.

**Fig 7:**
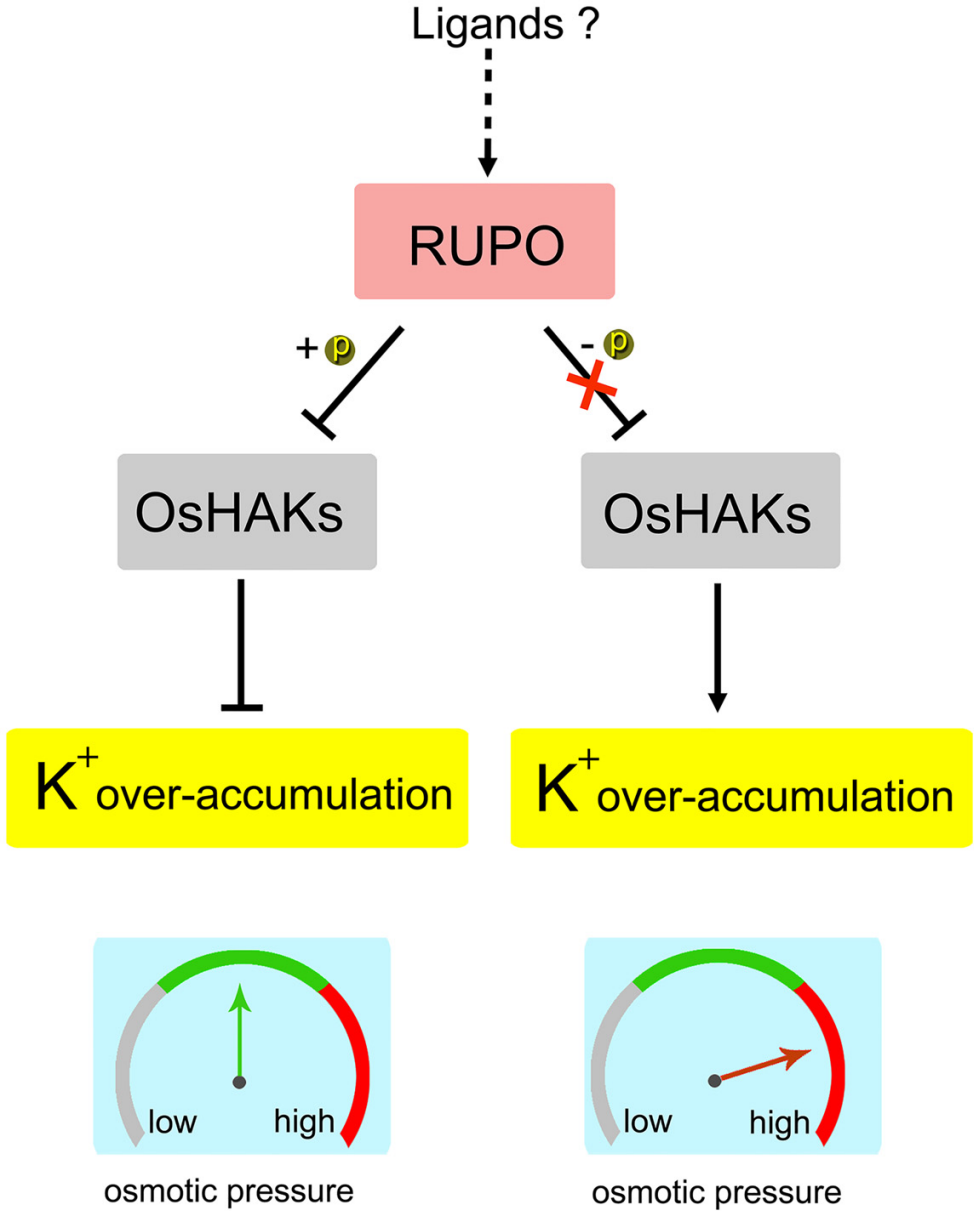
A proposed RUPO-potassium transporter signaling model to control pollen tube growth and integrity via regulating K^+^ homeostasis. The PM-localized RUPO is autophosphorylated, interacts with OsHAKs, and negatively regulates the high-affinity K^+^ transporter activity, finally establishing a K^+^ homeostasis. As the pollen tube enters the receptive synergid, unknown signals may cause change in RUPO phosphorylation, the inactivated RUPO no longer interacts with OsHAKs. The released OsHAKs cause K^+^ influx and over-accumulation in pollen tubes. High level potassium-mediated increase in turgor pressure leads to tube discharge.

Our data indicate the CrRLK1L–potassium transporter module as a novel CrRLK1L signaling pathway involved in PT growth and integrity. The proposed work model is as follows: during pollen maturity and PT growth, the PM-localized RUPO is autophosphorylated, then interacts with and negatively regulates K^+^ transporter activities to establish K^+^ homeostasis for pollen germination and PT growth (Fig 7). Studies showed that among functionally identified CrRLK1L members, RUPO is closest to THE1 of Arabidopsis, which can sense cell wall damage [39, 40]. Furthermore, synergids have high K^+^ concentration [52]. Therefore, with the arrival of PTs, the receptive synergid degenerates and releases K^+^, which may be transported into PTs. Impressively, we reveal that phosphorylation and dephosphorylation subtly control the interaction of RUPO with OsHAKs: phosphorylation established and dephosphorylation abolished the interaction. Together, these lines of evidence point to the possibility that the CrRLK1L-potassium module may involve in timely rupture and discharge of PTs. RUPO may sense signals from cell wall damage caused by the interaction of PTs and the receptive synergid, thereby releasing the inhibitory effect on K^+^ transporters, to lead to PT rupture and discharge via increasing K^+^ content in PTs. Admittedly, we cannot exclude the possibility that other K^+^ transport systems, such as shaker K^+^ channels may involve in this process. Further screening of RUPO-interacting K^+^ channels and signals from female tissues or from cell wall damage caused by male–female tissue interaction may be important to clarify this notion.

## Materials and Methods

### Plant materials and genotyping

Rice plants were grown under natural condition or in the greenhouse. T-DNA insertion mutant *rupo+/-* (*Oryza sativa* ssp. *japonica* cv. hwayoung) was obtained from the POSTECH database (http://cbi.khu.ac.kr/RISD_DB.html).

To obtain CRISPR-knockout lines, two sgRNA target sites were separately cloned into a modified pCambia1300 vector with sgRNA driven by OsU6 promoter, and optimized Cas9 was driven by ubiquitin promoter. Recombinant plasmids were transformed into *Agrobacterium tumefaciens* strain EHA105 and separately infected embryo-derived rice callus from hwayoung wild-type seeds. Transgenic rice seedlings were selected by using hygromycin. The mutant *rupo+/-* and its progeny plants were genotyped by using gene-specific primers and T-DNA border primers. CRISPR-knockout plants were genotyped by DNA sequencing.

For genomic DNA extraction and transforming of protoplasts, rice seeds were immersed in 75% ethanol (v/v) for 1 min and then in 2.5%(w/v) NaClO for 60 min. The surface-sterilized seeds were grown on half-strength Murashige & Skoog basal medium [53] supplemented with 1% sucrose and 0.8% agar, and kept at 28°C for 7–10 days in darkness before use.

Pollen at uninucleate microspore (UNM), BCP, and TCP stages were isolated from anthers. MPGs were collected from flowering panicles by using a modified vacuum cleaner. GPGs were obtained by pooling freshly collected MPGs in a liquid germination medium (4 mg/L H_3_BO_3_, 0.3 mM Ca(N0_3_)_2_.4H_2_O, 0.3 mg/L VB1, 10% PEG4000, 250 mM sucrose, pH 5.8) and incubating at 28~32°C for 15 min with gentle shaking.

### Molecular cloning of RUPO

Genomic DNA was extracted from etiolated rice seedlings by using a DNeasy plant mini kit (QIAGEN). Genomic *RUPO*, including a 2.8-kb promoter, the coding sequence, and 0.8-kb 3’UTR, was PCR-amplified with KOD-Plus-Neo DNA polymerase (TOYOBO) and cloned into the pEasy-blunt-zero vector (TransGen). To visualize the tissue expression pattern of *RUPO*::GUS, the 2.8-kb native RUPO promoter was fused to the pPLV15 vector via LIC cloning [54]. For genetic complementation, Genomic *RUPO* was fused into a modified pCambia1300 vector. The recombinant constructs were confirmed by DNA sequencing, then used for transforming rice callus induced from mature embryos.

### Expression analysis

For mRNA level examination, total RNA was isolated from various tissues by using the RNeasy Plant Mini Kit (QIAGEN). Isolated RNA was reverse transcribed by using SuperScript III reverse transcriptase (Invitrogen). Gene-specific primers were used for RT-PCR and qPCR (S2 Table). For protein level assay, proteins were separated by 12% SDS-PAGE. Proteins in a gel were electrophoretically transferred onto a PVDF membrane and detected with anti-RUPO antibody, which generated by immunizing rabbits (MBL, Beijing) with *E*. *coli*-expressed extracellular domain of RUPO.

### Transient expression assays

To generate *35S*::RUPOΔC-GFP and *Ubi*::RUPO-GFP constructs, RUPOΔC(1-500aa) and full-length RUPO were cloned into pA7-GFP and a modified pCambia1302 vector, respectively. For subcellular localization of OsHAKs-GFP, the coding sequences of OsHAK1/19/20 were fused to the C-terminal of GFP, which was driven by maize ubiquitin promoter. An amount of 10 μg of each plasmid DNA was precipitated onto submicron gold particles (1 μm gold). Particle bombardment of onion epidermal cells and lily pollen (*Lilium davidi* var. *unicdor cotton*) was carried out in a PDS-1000/He particle-delivery system with a target distance of 6.0 cm from the stopping plate at helium pressure of 1,100 p.s.i. After bombardment, onion epidermal cells were incubated on half-strength Murashige and Skoog basal salts containing 0.8% agar at 25°C overnight. For transient expression in lily pollen, 50 mg dry lily pollen was rehydrated at 4°C overnight and bombarded with 10 μg of plasmid. The bombarded pollen was incubated in germination medium [1 mM KCl, 1.6 mM H_3_BO_3_, 0.5 mM CaCl_2_, 15% sucrose (w/v), pH5.8] at 26°C for 6 h with gentle shaking at 60 rpm before observation. Fluorescence images were acquired by laser confocal microscopy (Olympus FV1000MPE).

### Pollen assays

For Alexander staining, mature anthers before anthesis were cut and immersed in Alexander’s solution [55] overnight at room temperature. Stained anthers were crushed, and pollen grains were observed under bright field microscopy. For 4’,6-diamidino-2-phenylindole (DAPI) staining, mature pollen grains were fixed in Carnoy's solution (30% chloroform, 10% acetic acid, 57% ethanol) for 2 h at room temperature and stained in a 1 μg/mL DAPI solution (50% glycerol, 140 mM NaCl, 2.7 mM KCl, 10 mM Na_2_HPO_4_, 1.8 mM KH_2_PO_4_, pH 7.3) for 10 min at room temperature in the dark. DAPI-stained pollen grains were observed under UV light. For GUS staining, rice florets were harvested and fixed in 90% (v/v) acetone at room temperature for 1 h. After washing with 0.1M K_2_HPO_4_ (pH 8.5), these florets were immersed in 1 mM K4[Fe(CN)6], 1 mM K3[Fe(CN)6], 1 mM X-Gluc, 0.1% Triton X-100, 10 mM EDTA, 100 mM K_2_HPO_4_ (pH 8.5) and incubated at 37°C overnight in the dark. Pollen coat was observed under a scanning electric microscope (HITACHI S-800) and ultrastructure was observed under a transmission electron microscopy (JEM-1230) as described [56].

To examine pollen germination and burst rate in vitro, pollen grains from dehisced anthers were directly shed onto a solid germination medium [0.8% low gelling temperature agarose II (Amresco), 4 mg/L H_3_BO_3_, 0.3 mM Ca(N0_3_)_2_.4H_2_O, 0.3 mg/L VB1, 10% PEG4000, 250 mM sucrose, pH 5.8] which was layered onto a glass slide (Leica). Briefly, the agarose medium was mixed and molten by heating, then 3×2 cm pads were drawn by using 0.5 mL of molten agarose medium on slides. The slides were left to cool to room temperature, placed in 150-mm tissue culture dishes (Corning) and used within 30 min. Immediately after floret opening, florets were gently excised by using scissors, and pollen grains were shed onto the solid germination medium. After 10-min incubation at 28~30°C, the slides were observed by microscopy with a 10×objective and images of 3 randomly chosen fields were photographed. Only the images with more than 15 pollen grains and with pollen germination rate > 50% were used for subsequent pollen germination rate or integrity analysis.

In vivo PT growth was observed by aniline blue staining. At 6 h after artificial or natural pollination, rice pistils from mutant and wild type plants were cut, and fixed in Carnoy's solution overnight. The pistils were then washed 5 times with water, softened by incubating in 1 mol /L NaOH at 55°C for 30 min and stained in 0.1% (w/v) aniline blue (in 0.1 M K_2_HPO_4_, pH 8.5) at room temperature for 4–16 h in the dark. Fluorescent images were observed with a ZEISS microscope (Axio Imager A1) under UV light.

### Autophosphorylation assay

cDNAs encoding full-length RUPO (29–845 aa, without the signal peptide) and the intracellular domain (515–845 aa) were PCR-amplified. The cDNA encoding the kinase-deficient version (29–845 aa with K543R mutation) was prepared with site-directed mutagenesis to change the essential K at 543 to R in the conserved ATP binding domain. The cDNAs were cloned in-frame into pETMALc-H vector at *Bam*H I and *Hin*d III sites, sequenced and transformed into BL21 cells (Rosetta DE3) to generate maltose-binding protein (MBP)-tagged proteins.

BL21 cells were grown in LB medium at 37°C to OD_600_ of 0.6, then treated with 0.1 mM IPTG at 25°C for 4 h. Cells were harvested by centrifugation, resuspended in cold HEPES buffer (50 mM HEPES, 200 mM NaCl, pH 7.4), supplemented with 1 mM DTT, 1 mg/mL lysozyme, and 1 mM PMSF, and lysed by sonication. Lysate was centrifuged at 15,000 g at 4°C for 15 min, and target proteins in the supernatant were purified with Amylose resin (New England Biolabs). MBP tag was removed by adding approximately 10 U thrombin (GE Healthcare) per mg recombinant proteins, and on-column–cleaved overnight at room temperature. Flowthrough containing target proteins was passed through a HiTrap Benzamidine FF column (GE Healthcare) to remove residual thrombin.

In vitro kinase assay was carried out in a 30-μL reaction mixture containing 50 mM HEPES, pH 7.4, 10 mM MnCl_2_,10 mM MgCl_2_, 1 mM DTT, 20 μM unlabeled ATP, 10 μCi [γ-32P]ATP (3000 Ci/mmol, 10 mCi/mL, Perkin Elmer) and 6 μg each protein. Reactions were stopped after 30 min at room temperature by adding 10 μL 4×SDS-PAGE loading buffer and heating at 95°C for 5 min. Proteins were separated by 10% SDS-PAGE. The gel was stained with Coomassie brilliant blue, destained, dried and exposed to x-ray film.

### Yeast two hybrid assay

The pGBKT7 and pGADT7-Rec vectors (Clontech) were used to construct bait and prey cDNA library, respectively. The DNA fragment encoding the intracellular domain (515–845 aa, without JM) of RUPO was ligated in-frame into pGBKT7 at *Nde*I and *Eco*RI sites to produce a bait construct. The bait plasmid was transformed into yeast strain Y2HGold, and bait expression was confirmed by western blot assay with anti-myc antibody. Total mRNAs from in vivo-germinated pollen were prepared by using the RNeasy plant mini kit (QIAGEN) and on-column–digested with DNase I (QIAGEN). cDNA were synthesized (CDS III primer) and co-transformed with linearized pGADT7-Rec vector into yeast strain Y187 by using the Mate & Plate library system (Clontech). The yeast cells were then spread on 100×150 mm SD/-Leu agar plates. The library screening was performed by incubating 1 mL pooled library (Y187) with bait strain (Y2HGold) at 30°C for 22 h with gentle shaking at 40 rpm. About 5 million diploids were screened with a mating efficiency of 2.8%. Mating diploids were first spread on SD/-His/-Leu/-Trp plates, and the selected colonies were transferred onto SD/-Ade/-His/-Leu/-Trp plates. Only colonies that grew on SD/-Ade/-His/-Leu/-Trp plates and turned blue in the presence of x-α-Gal were used for plasmid rescue and DNA sequencing.

### Pull down assay

To express the C-terminal domains of OsHAKs, PCR products corresponding to the C-terminal domains (683–792 aa) were cloned into pETMALc-H at *Bam*H I and *Hin*d III sites to generate MBP-tagged proteins. BL21 cells (Rosetta DE3) harboring recombinant plasmids were grown in LB medium at 37°C to OD_600_ 0.6, then treated with 0.1 mM IPTG at 20°C for 7 h. The resultant cells were harvested by centrifugation at 5,000 g at 4°C for 10 min, suspended in washing buffer (20 mM Tris-HCl, 200 mM NaCl, 1 mM DTT, 1 mg/ml lysozyme, and 1 mM PMSF, pH 7.4), and lysed by sonication. The lysate was centrifuged at 12,000 g at 4°C for 15 min, and MBP-tagged proteins in the supernatant were purified with Amylose resin (New England Biolabs).

For expression of GST-RUPO-C, PCR product corresponding to 515~845 aa residues of RUPO was cloned into pGEX-4T-1 at *Bam*H I and *Eco*R I sites, sequenced and transformed into BL21 cells (DE3). The cells were grown in LB medium at 37°C to OD_600_ 0.6, treated with 0.1 mM IPTG at 20°C for 10 h and harvested by centrifugation. The pelleted cells were suspended in PBS (140 mM NaCl, 2.7 mM KCl, 10 mM Na_2_HPO_4_, and 1.8 mM KH_2_PO_4_, 1 mM DTT, 1 mg/mL lysozyme, and 1 mM PMSF, pH 7.5), then lysed by sonication. The lysate was centrifuged at 12,000 g at 4°C for 15 min, and GST-tagged proteins in the supernatant were purified with glutathione sepharose 4B beads (GE).

Protein interaction was tested by in vitro pull-down assay. Briefly, 100 μg of bait proteins (MBP-tagged proteins or MBP) were incubated with 20 μL amylose resin (NEB) in washing buffer (20 mM Tris-HCl, 150 mM NaCl, 1 mM DTT, pH 7.5). The resin was washed 3 times with washing buffer and the bead–protein complexes were incubated with 15 μg prey proteins (GST-RUPO-C or GST) in wash buffer at 4°C for 3 h, and washed again for 5 times. The pull-down samples were eluted with 1×SDS loading buffer and analyzed with anti-GST and anti-MBP antibodies.

### Co-immunoprecipitation (Co-IP) assay

Approximately 2×10^6^ rice protoplasts co-transformed with indicated plasmids were lysed with 400 μL extraction buffer [10 mM HEPES, 100 mM NaCl, 1 mM EDTA, 10% (v/v) glycerol, 0.5% Triton X-100, pH 7.5] supplemented with 1×protease inhibitor cocktail (Roche). The samples were briefly sonicated and centrifuged at 5,000 g at 4°C for 5 min. The supernatant was incubated with 20 μL anti-FLAG agarose (MBL) at 4°C for 3 h with gentle shaking. The beads were collected and washed 5 times with washing buffer (10 mM HEPES 100 mM NaCl, 1 mM EDTA, 10% glycerol, and 0.1% Triton X-100, pH 7.5), boiled in 1×SDS loading buffer and examined by western blot analysis with anti-GFP or anti-FLAG antibody.

### LC-Q-TOF MS/MS identification of phosphorylation sites

Briefly, 12 μg MBP-RUPO was incubated in kinase buffer (50 mM HEPES, pH 7.6, 10 mM MnCl_2_, 10 mM MgCl_2_, 1 mM DTT, 100 μM ATP) at 30°C for 30 min, and autophosphorylation was stopped by adding 0.1% RapiGest SF (Waters). Protein samples were reduced in 5 mM DTT at 60°C for 30 min and alkylated with 15 mM Iodoacetamide at room temperature in the dark for 30 min. The resultant proteins were enzymatically digested by adding 1:20 (w/w) trypsin (Sigma) or endoproteinase Glu-C (Sigma) and incubated overnight at 37°C. The digestion was terminated by adding 0.5% trifluoroacetic acid, and the mixture was incubated again at 37°C for 40 min. After centrifugation at 20,000g for 10 min, the supernatant was used for phosphopeptide enrichment by using a Titansphere Phos-TiO_2_ kit (GL Sciences). The enriched peptides were divided in half and dried in SpeedVac (Thermo). One half was treated with alkaline phosphatase before MS analysis, and the other half was used for MS analysis directly. LC-ESI-MS/MS analysis involved a TripleTOF 5600 mass spectrometer (AB SCIEX) equipped with a NanoSpray III source. Separation of peptides was achieved on an Eksigent 1D Plus Ultra LC system incorporating a C18-ChromXP column (0.075×150 mm, 3 μm, 120 Ǻ, Eksigent). Peptides were on-line desalted for 10 min at a flow rate of 2 μL/min by using an in-house packed C18-Trap column (0.1×2.5 mm, IntegraFrit ProteoPep II) and eluted at 0.3 μL/min with a 90-min linear gradient of acetonitrile/H_2_O containing 0.1% formic acid. MS-TOF scans were recorded for 0.25 s, and the 40 most-abundant ions in the survey spectra were automatically selected for collision-induced dissociation. The MS/MS raw data were searched against the *Oryza sativa* database (NCBInr) by using ProteinPilot 4.5 (Paragon method) and the search parameters: Cys. Alkylation: Iodoacetamide; Special Factors: Phosphorylation emphasis; ID Focus: Biological modifications; Search Effort: Thorough; Detected Protein Threshold [Unused ProtScore (Conf)] >:0.05 (10.0%). For Mascot search, the data file was converted to a Mascot generic format with use of ProteinPilot 4.5, then searched by using an in-house Mascot server (version 2.4). Mascot search parameters were set to mass tolerance of 0.05 Da for the precursor ions and 0.1 Da for the fragment ions. One trypsin/Glu-C missed cleavage site was allowed. Peptides with +2, +3 and +4 net charge were checked. Carbamidomethylation of cysteine was set as a fixed modification, and oxidation of methionine and phosphorylation were set as variable modifications. Both Paragon and Mascot searches involved false discovery rate analysis, and a threshold corresponding to a false discovery rate of ≤ 1% was used to determine confident peptide matches.

### Inductively coupled plasma atomic emission spectroscopy (ICP-OES)

To determine the potassium level in MPGs, MPGs from the wild type and bi-allelic CRISPR plants were collected and dried in an incubator at 40°C for 1 h. About 2 mg dried MPGs were placed in 2 mL microtubes containing 1mL of 2% (v/v) nitric acid and 30 glass beads (2 mm, Merck), and broken by using a FastPrep-24 homogenizer (MP Biomedicals) at 6.5 m/s for 60 s. The control tubes contained the same amount of 2% nitric acid and glass beads but without MPGs. The homogenates were centrifuged twice at 12,000 g for 15 min. In total, both the samples and control were extracted with 2% nitric acid for 3 times. The supernatants were pooled and analyzed by using an iCAP 6300-ICP-OES CID Spectrometer (Thermo Scientific). Potassium standard (Cat. 96665) for determination was purchased from Sigma-Aldrich.

### Yeast complementation

The coding sequences of *OsHAK1*, *OsHAK19*, and *OsHAK20* were cloned into yeast expression vector YES3 and transformed into R5421 strain (*trk1*Δ *trk2*Δ), which lacks two PM potassium transporters [57,58]. The transformants were incubated overnight in SD/-Trp medium supplemented with 100 mM KCl. Yeast cells were harvested by centrifugation and resuspended in H_2_O to an OD_600_ of 0.8. 1/10 serial diluted cells were dropped on arginine phosphoric (AP) medium [59] with different K^+^ concentrations. These plates were incubated at 30°C for 3 d.

### Accession numbers

Sequence data from this article can be found in the Rice Genome Annotation Project databases under accession nos. LOC_Os06g03610 (*RUPO*), LOC_Os04g32920 (*OsHAK1*), LOC_Os02g31910 (*OsHAK19*), LOC_Os02g31940 (*OsHAK20*).

## Supporting Information

S1 Fig Phenotypic observation of T-DNA insertional mutant *rupo+/-*. (A) Images of wild-type, *rupo+/-* and complementation plants. Scale bars, 10 cm. (B) Images of rice florets harvested just before dehiscence. Scale bars, 2 mm. (C) Southern blot analysis of *rupo+/-*. (D) PCR genotyping of *rupo+/-* and complementation plants.
(TIF)

S2 Fig Expression pattern of *RUPO*::GUS. (A) A rice floret, (B) pistil, (C) anther, and (D) pollen grains. *GUS* gene was driven by a 2.8-kb *RUPO* promoter. The rice florets were harvested when the spike was fully emerged but without dehiscence. Scale bars, 100 μm.
(TIF)

S3 Fig Anti-RUPO polyclonal antibodies specifically reacted with native RUPO. IP, Immunoprecipitation of native RUPO by anti-RUPO. GPG, crude membrane proteins extracted from germinated pollen grains. MPG, crude membrane proteins extracted from mature pollen grains. Minus sign denotes pre-immune serum; plus sign denotes anti-RUPO. IgG HC denotes heave chains of immunoglobulin G.
(TIF)

S4 Fig IP-MS/MS identification of native RUPO. Peptide sequences identified by MS/MS were highlighted (green, underlined). Note that native RUPO is lack of signal peptide (1~28 aa).
(TIF)

S5 Fig Morphological observation of mature pollen grains. (A,B) DAPI staining of wild-type (A) and *rupo*+/- pollen (B). (C,D) Alexander staining of wild-type (C) and *rupo*+/- pollen (D). (E,F) Scanning electron microscopy of wild-type (E) and *rupo*+/- pollen (F). (G to J) Transmission electron microscopy of wild-type (G,H) and *rupo*+/- pollen (I,J). Scale bars, 50 μm in (A) to (F), and 2 μm in (G) to (J).
(TIF)

S6 Fig Genotyping of CRISPR plants. (A) Schematic of RUPO Protein. Arrowheads denote the two target sites *sg1* and *sg2* on the extracellualr domain of RUPO. The target sequences (green) and protospacer-adjacent motif sequence (red) are indicated. SP, signal peptide; TM, Transmembrane domain. (B) sgRNA:Cas9-induced *RUPO-sg1* and *RUPO-sg2* mutations in transgenic rice plants. The nucelotide insertions are represented by lower case letters, and deletions are represented by dash marks(-).
(TIF)

S7 Fig CRISPR mutants show normal vegetative and reproductive growth. (A) Plant phenotype of wild-type, (B) bi-allelic homozygous CRISPR mutant, and (C) mono-allelic heterozygous CRISPR mutant. Scale bars, 10 cm. (D-I) Images of rice florets harvested just before dehiscence. Scale bars, 1mm. (J,K,L) Alexander staining of pollen grains. Scale bars, 100 μm. (M,N,O) DAPI staining of pollen grains. Scale bars, 50 μm. (P) A wild-type anther before dehiscence. (Q) A wild-type anther after dehiscence. (R) A bi-allelic anther before dehiscence. (S) A bi-allelic anther after dehiscence. (T) A mono-allelic anther before dehiscence. (U) A mono-allelic anther after dehiscence. Bars, 200 μm.
(TIF)

S8 Fig Pollen tubes from homozygous bi-allelic mutant burst during in vitro germination. (A,B,C) In vitro germination assays of wild-type (A), mono-allelic CRISPR (*rupo-/+*) (B) and bi-allelic CRISPR (*rupo-/-*) pollen (C). Red arrowheads indicate ruptured pollen tubes. Scale bars, 100 μm. (D) Quantification of pollen germination rate and percentage of pollen tube integrity. The results are presented as mean±s.e.. 588 wild-type pollen (from 6 plants), 280 mono-allelic pollen (from 2 plants) and 444 bi-allelic pollen (from 3 plants) were used for statistical analysis.
(TIF)

S9 Fig Percentage of germinated pollen grains in the aniline blue-stained stigma. After aniline blue staining, residual germinated pollen grains (GPGs) and non-germinated pollen grains that attached to the stigma were counted separately. 544 wild-type pollen and 138 *rupo* pollen were used for statistical analysis. The results are presented as mean±s.e..
(TIF)

S10 Fig Bi-allelic pistil is fertile. (A) Wild-type was used as pollen donor, bi-allelic as pollen receptor. White arrows indicate florets that produce seeds (B) Bi-allelic was used as pollen donor, wild-type as pollen receptor. Note that all glumes are empty. (C) Image of a bi-allelic spike 30 days after self-pollination. (D) Image of a wild-type spike 30 days after self-pollination. (E) Aniline blue staining shows a wild-type pollen tube targeting a bi-allelic micropyle. (F) A bi-allelic pollen grain germinated in a wild-type pistil but failed to reach the ovule. Scale bars, 2 cm in (A-D), 200 μm in (E,F).
(TIF)

S11 Fig Protein sequence analysis of OsHAKs. Protein sequence alignment of OsHAK1, OsHAK19 and OsHAK20 was performed with Clustal Omega program. Asterisks indicate identical amino acids. The dots indicate similar amino acids. Note that OsHAK1 contains an extra 35 amino acid residues (678-712aa) in the C-terminus.
(TIF)

S12 Fig Topology prediction of OsHAK1. OsHAK1 protein contains 792 amino acid residues and 12 transmembrane domains, with N- and C-terminus inside the cytoplasm. Topology and transmembrane domains were predicted by using HMMTOP 2.1 software.
(TIF)

S13 Fig Yeast two-hybrid analysis of RUPO interaction with different C-tails of OsHAKs. The C-tails of OsHAK1, OsHAK6, OsHAK17, OsHAK19, OsHAK20, OsHAK23, OsHAK25, and OsHAK26 were cloned from total RNA of mature pollen grains. The 77 aa length (A) and 110 aa length (B) C tails were used for the interaction assays.
(TIF)

S14 Fig Subcellular localization of OsHAK1, OsHAK19 and OsHAK20. (A to F) Subcellular localization of the *Ubi*::OsHAKs-GFP fusion proteins in onion epidermal cells. (A) Single confocal section and (D) bright-field image of the cell bombarded with *Ubi*::OsHAK1-GFP plasmid. (B) Single confocal section and (E) bright-field image of the cell bombarded with *Ubi*::OsHAK19-GFP. (C) Single confocal section and (F) bright-field image of the cell transformed with *Ubi*::OsHAK20-GFP. (G to L) Transient expression of *Ubi*::OsHAK1-GFP (G,J), *Ubi*::OsHAK19-GFP (H,K), and *Ubi*::OsHAK20-GFP (I,L) in lily pollen tubes. Scale bars, 20 μm.
(TIF)

S15 Fig Complementation assay of OsHAK1/19/20 in a K^+^ uptake deficient yeast strain. R5421 strain were transformed with OsHAK1/19/20 or expression vector pYES3 (control), and the transformants were dropped onto AP medium in 1:10 serial dilutions.
(TIF)

S16 Fig Effects of K^+^ on pollen germination and tube integrity in vitro. The original pollen germination medium contained 50 μm K^+^. The KCl concentrations are indicated. Scale bars, 50 μm.
(TIF)

S1 Table Identification of 10 *in vitro* phosphorylation sites of RUPO. ^a^The amino acid sequence of phosphorylated peptides, where Ser/Thr phosphorylation and carbamidomethyl modification are denoted by [Pho],[CAM] respectively. ^b^The experimentally measured monoisotopic mass for the precursor ion fragmented. ^c^The difference in mass between the precursor MW and the theoretical MW of the matching peptide sequence. ^d^Peptide confidence given by ProteinPilot 4.5. ^e^Mascot score. Individual ion score >34 (for trypsin) or score >29 (for Glu-C) means the probability of random match is less than 0.05. In total, the MS/MS data covered > 96.95% amino acid sequence of the intracellular domain, including all Ser/Thr/Tyr residues except for Tyr- 588.
(PDF)

S2 Table List of primers. (PDF)

S1 Movie In vitro germination assay of wild-type pollen. (AVI)

S2 Movie In vitro germination assay of *rupo* pollen. (AVI)

S3 Movie Dynamics of RUPO-GFP foci at the tip region of lily pollen tube. RUPO-GFP was excited by a 473-nm laser. Fluorescence signals were collected by using VA-TIRFM, which consisted of an inverted microscope(IX-71, Olympus), TIRFM illumination module(IX2-RFAEVA-2, Olympus) and an Olympus 100× PlanApo oil objective.
(AVI)

S4 Movie Dynamics of RUPO-GFP foci at the shank region of lily pollen tube. (AVI)
