# Supplementary figures and images for: Receptor-Like Kinase RUPO Interacts with Potassium Transporters to Regulate Pollen Tube Growth and Integrity in Rice
Source: PLoS Genet. 2016 Jul 22;12(7):e1006085. doi: 10.1371/journal.pgen.1006085 (PMC4957769; doi:10.1371/journal.pgen.1006085)

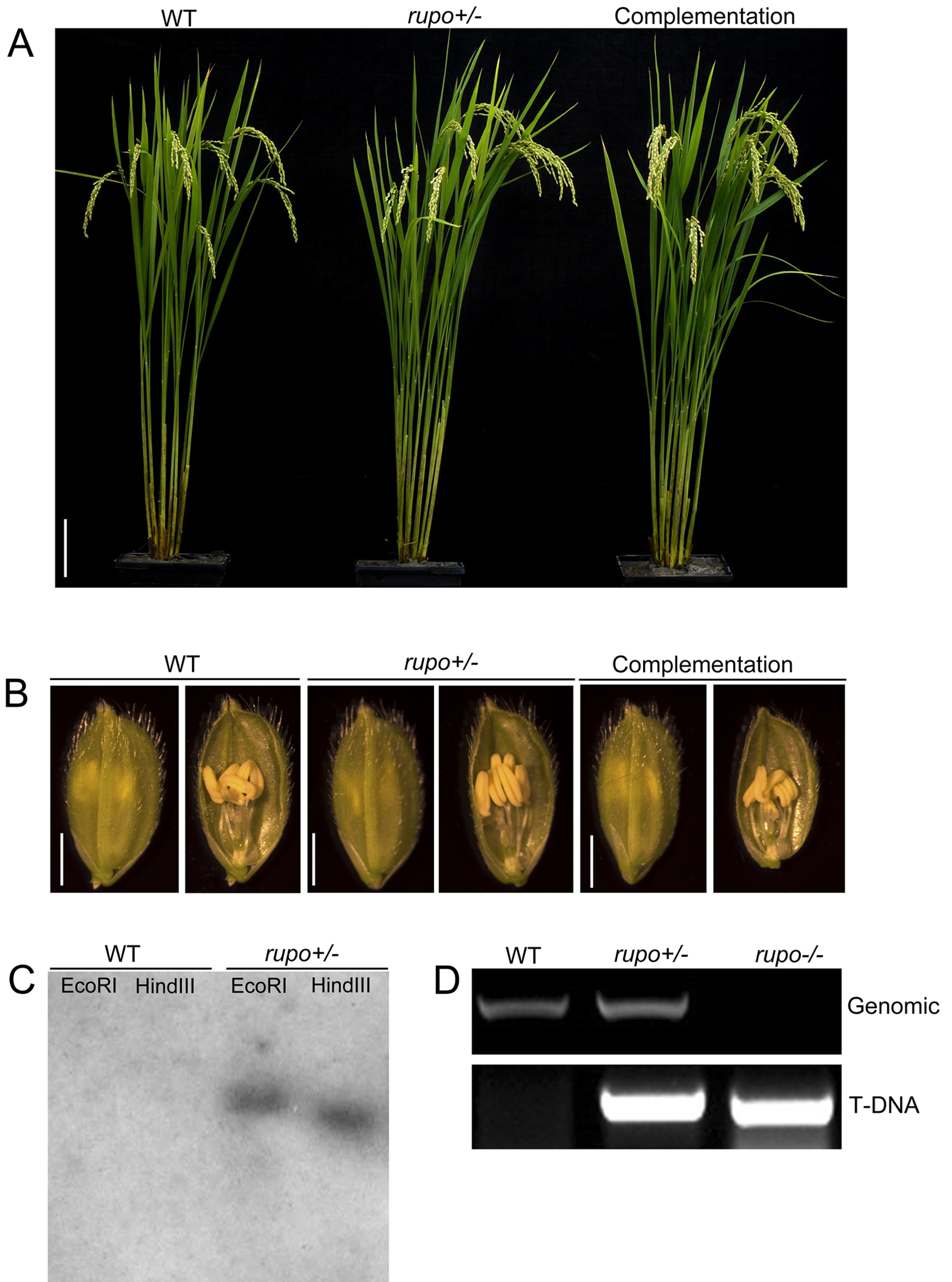

Supplement: S1 Fig — (A) Images of wild-type, rupo+/- and complementation plants. Scale bars, 10 cm. (B) Images of rice florets harvested just before dehiscence. Scale bars, 2 mm. (C) Southern blot analysis of rupo+/-. (D) PCR genotyping of rupo+/- and complementation plants. (TIF) [file pgen.1006085.s001.tif]

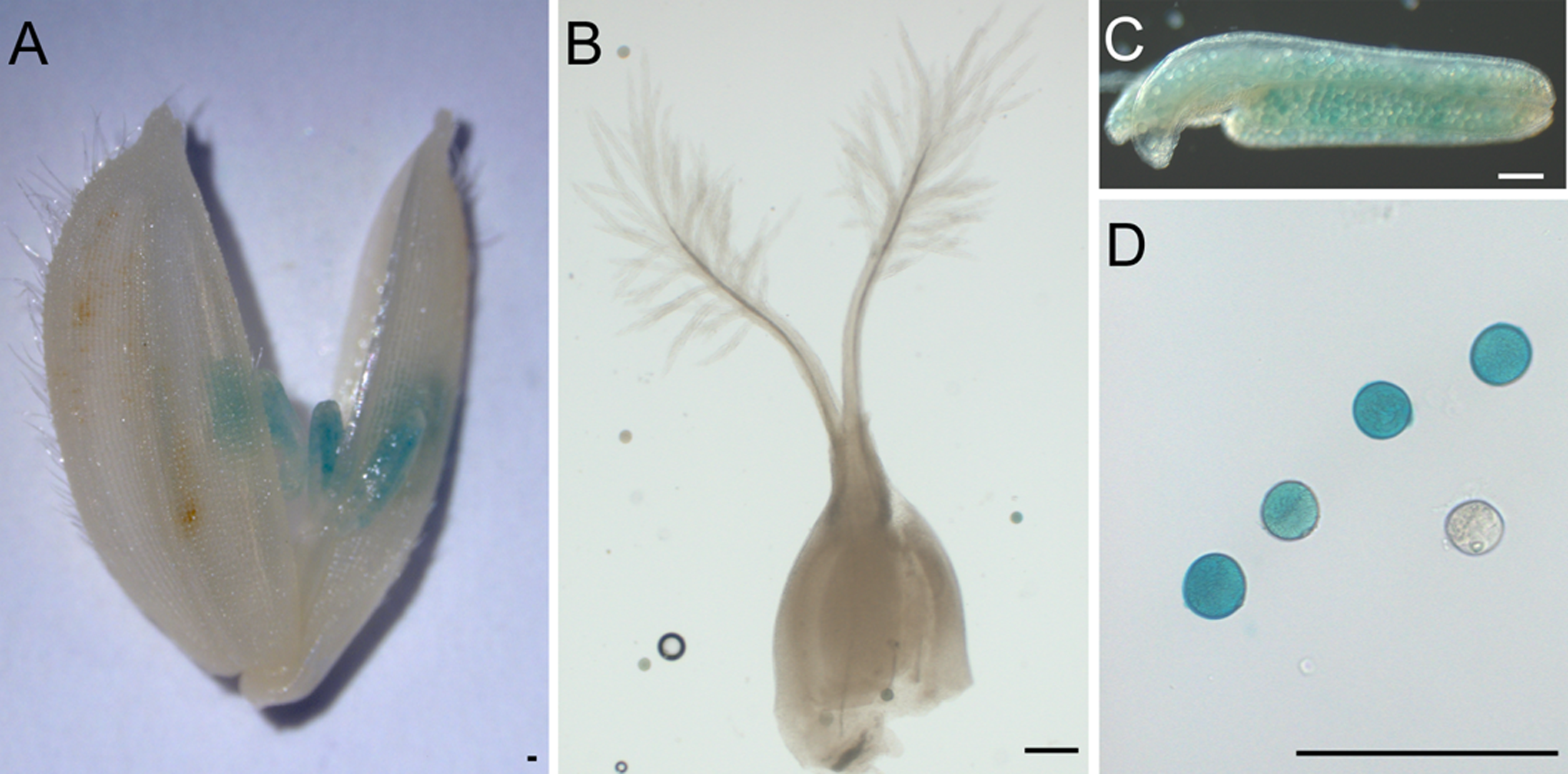

Supplement: S2 Fig — (A) A rice floret, (B) pistil, (C) anther, and (D) pollen grains. GUS gene was driven by a 2.8-kb RUPO promoter. The rice florets were harvested when the spike was fully emerged but without dehiscence. Scale bars, 100 μm. (TIF) [file pgen.1006085.s002.tif]

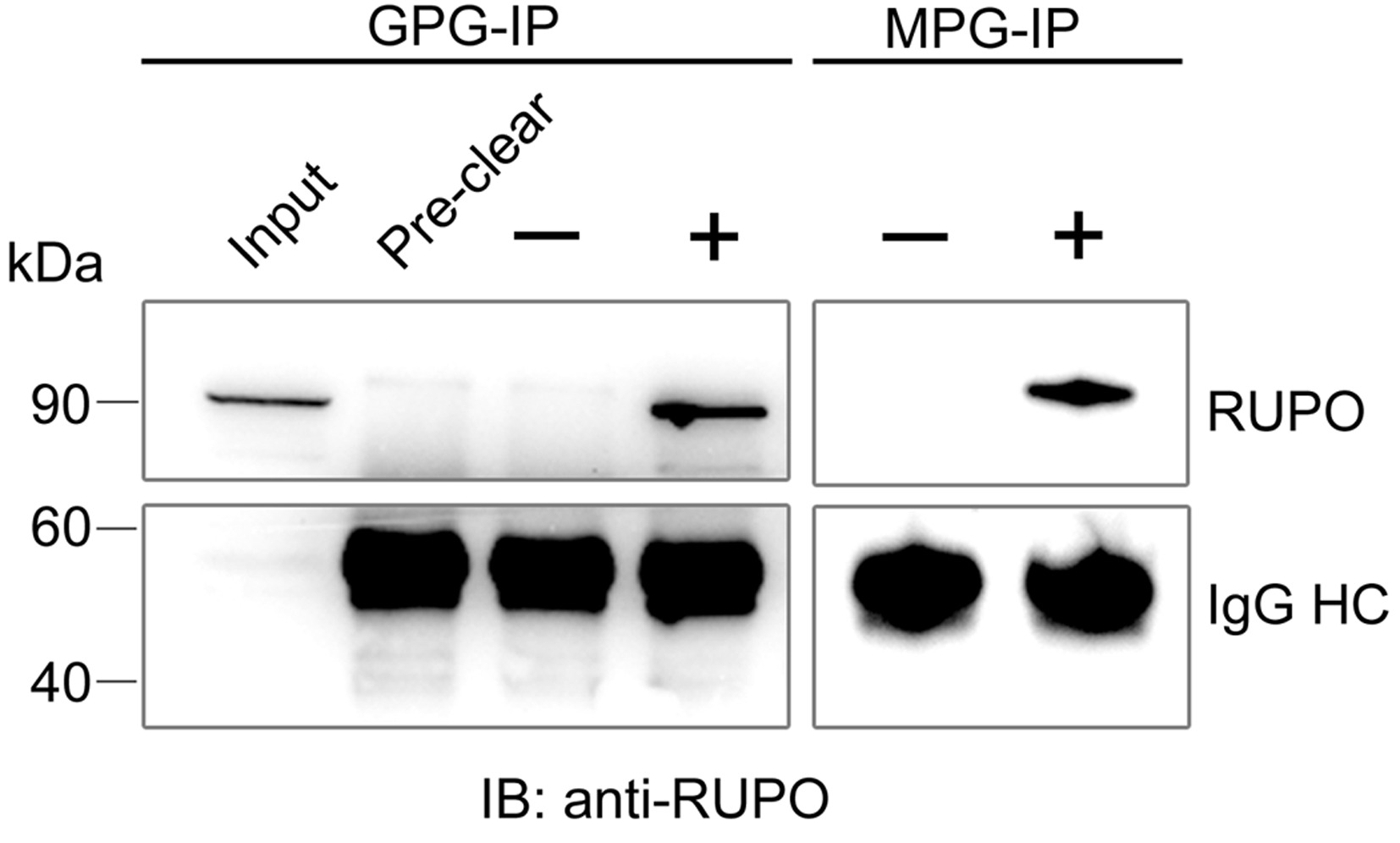

Supplement: S3 Fig — IP, Immunoprecipitation of native RUPO by anti-RUPO. GPG, crude membrane proteins extracted from germinated pollen grains. MPG, crude membrane proteins extracted from mature pollen grains. Minus sign denotes pre-immune serum; plus sign denotes anti-RUPO. IgG HC denotes heave chains of immunoglobulin G. (TIF) [file pgen.1006085.s003.tif]

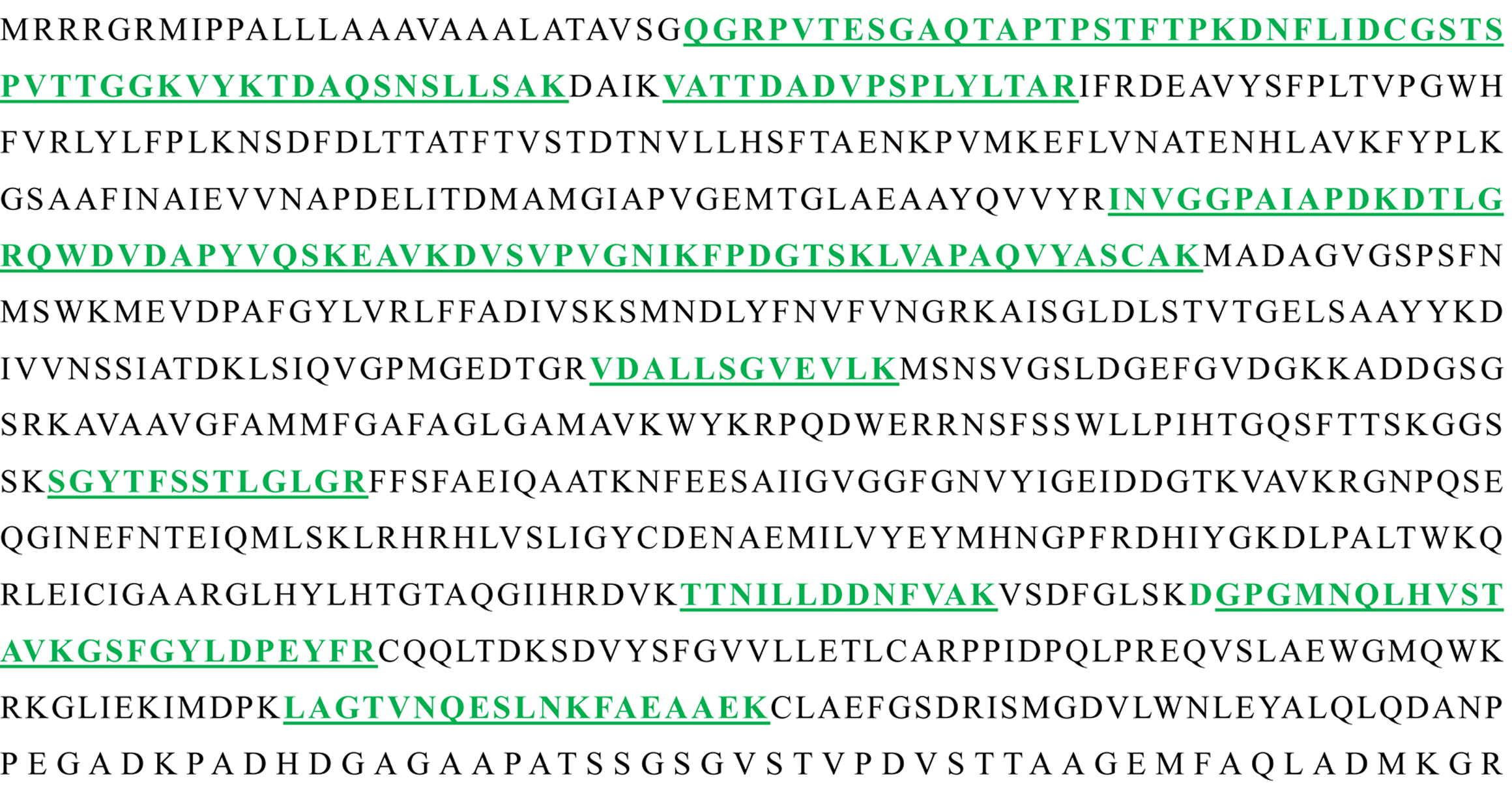

Supplement: S4 Fig — Peptide sequences identified by MS/MS were highlighted (green, underlined). Note that native RUPO is lack of signal peptide (1~28 aa). (TIF) [file pgen.1006085.s004.tif]

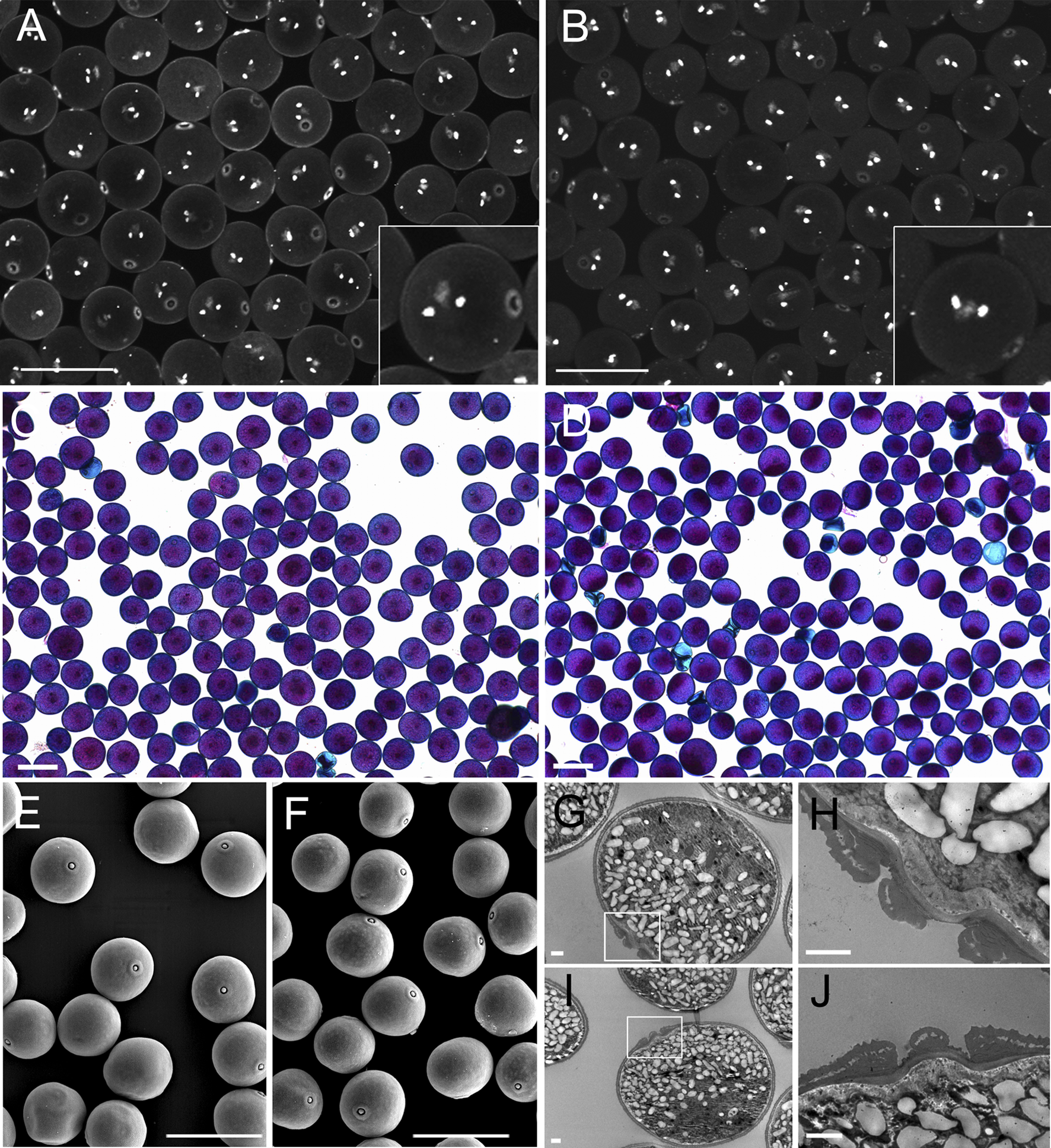

Supplement: S5 Fig — (A,B) DAPI staining of wild-type (A) and rupo+/- pollen (B). (C,D) Alexander staining of wild-type (C) and rupo+/- pollen (D). (E,F) Scanning electron microscopy of wild-type (E) and rupo+/- pollen (F). (G to J) Transmission electron microscopy of wild-type (G,H) and rupo+/- pollen (I,J). Scale bars, 50 μm in (A) to (F), and 2 μm in (G) to (J). (TIF) [file pgen.1006085.s005.tif]

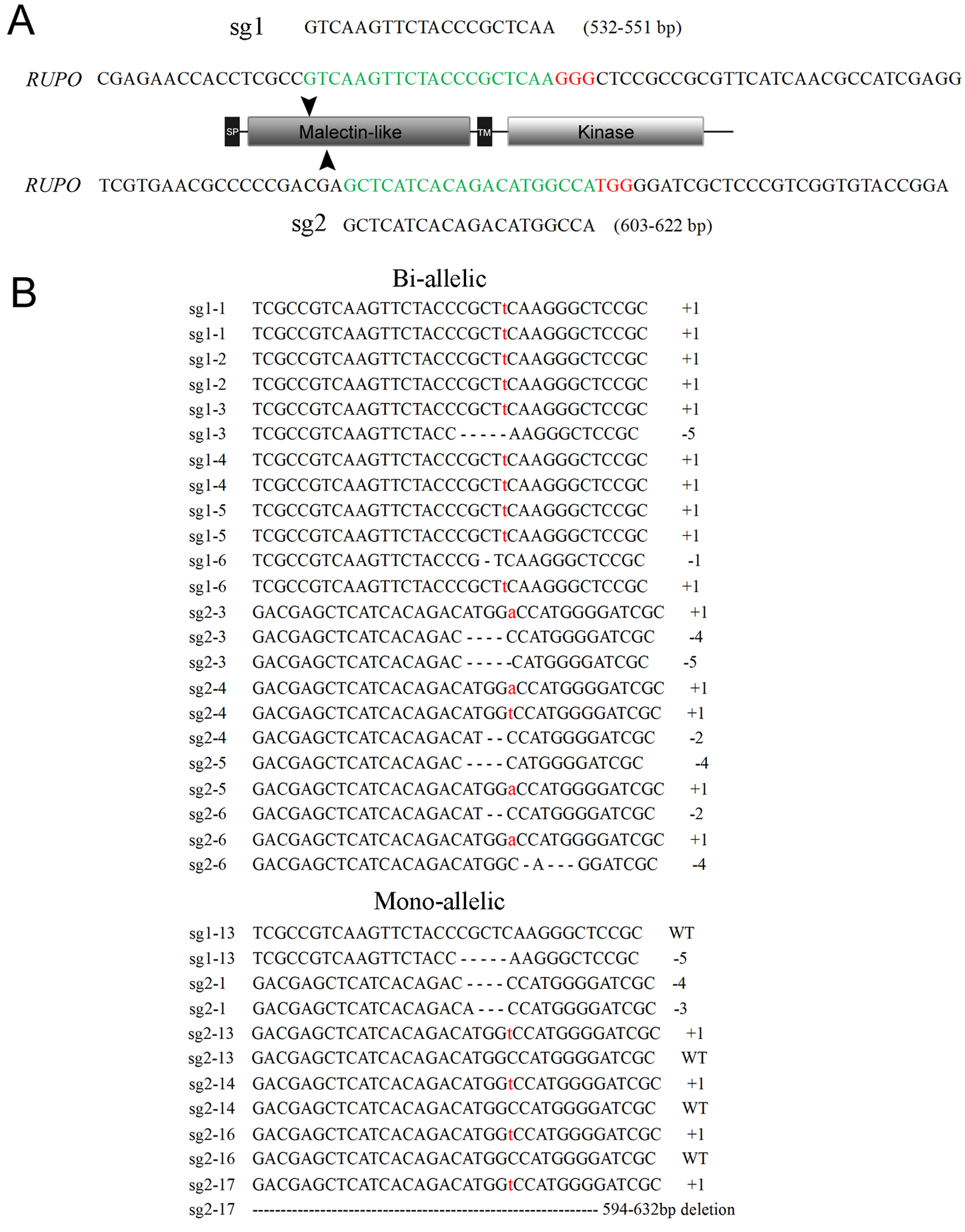

Supplement: S6 Fig — (A) Schematic of RUPO Protein. Arrowheads denote the two target sites sg1 and sg2 on the extracellualr domain of RUPO. The target sequences (green) and protospacer-adjacent motif sequence (red) are indicated. SP, signal peptide; TM, Transmembrane domain. (B) sgRNA:Cas9-induced RUPO-sg1 and RUPO-sg2 mutations in transgenic rice plants. The nucelotide insertions are represented by lower case letters, and deletions are represented by dash marks(-). (TIF) [file pgen.1006085.s006.tif]

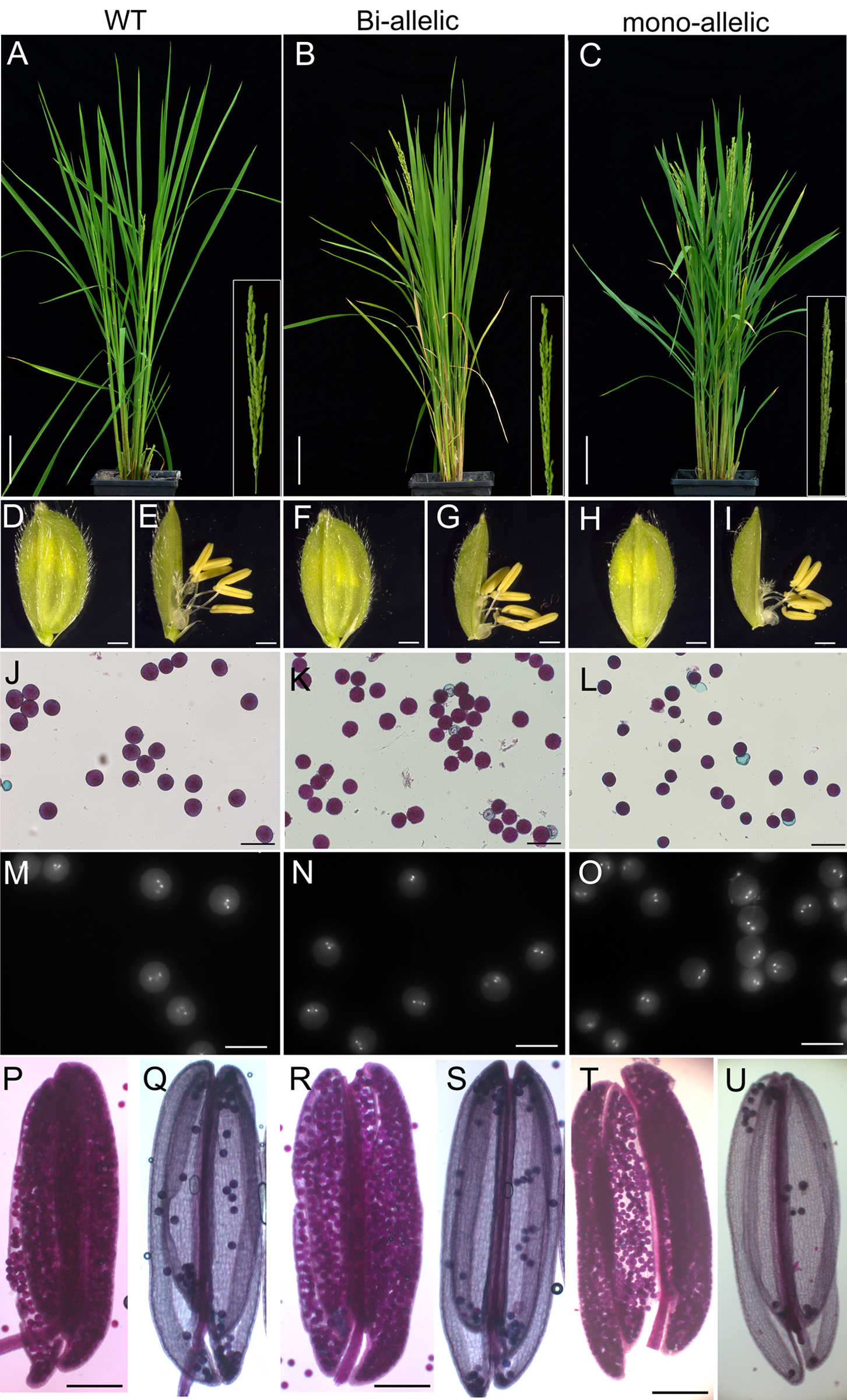

Supplement: S7 Fig — (A) Plant phenotype of wild-type, (B) bi-allelic homozygous CRISPR mutant, and (C) mono-allelic heterozygous CRISPR mutant. Scale bars, 10 cm. (D-I) Images of rice florets harvested just before dehiscence. Scale bars, 1mm. (J,K,L) Alexander staining of pollen grains. Scale bars, 100 μm. (M,N,O) DAPI staining of pollen grains. Scale bars, 50 μm. (P) A wild-type anther before dehiscence. (Q) A wild-type anther after dehiscence. (R) A bi-allelic anther before dehiscence. (S) A bi-allelic anther after dehiscence. (T) A mono-allelic anther before dehiscence. (U) A mono-allelic anther after dehiscence. Bars, 200 μm. (TIF) [file pgen.1006085.s007.tif]

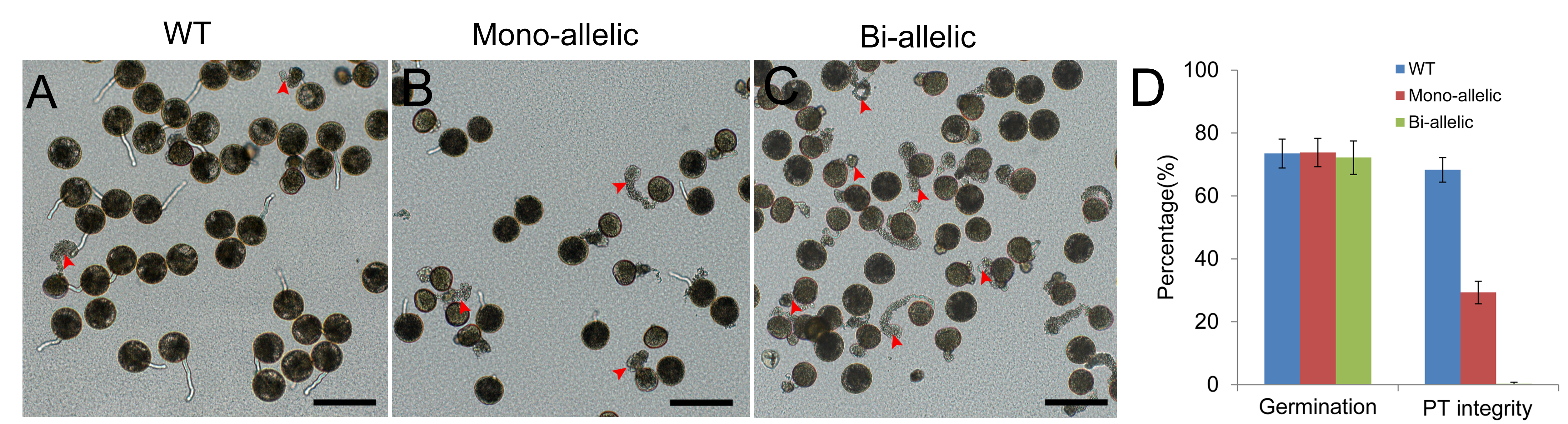

Supplement: S8 Fig — (A,B,C) In vitro germination assays of wild-type (A), mono-allelic CRISPR (rupo-/+) (B) and bi-allelic CRISPR (rupo-/-) pollen (C). Red arrowheads indicate ruptured pollen tubes. Scale bars, 100 μm. (D) Quantification of pollen germination rate and percentage of pollen tube integrity. The results are presented as mean±s.e.. 588 wild-type pollen (from 6 plants), 280 mono-allelic pollen (from 2 plants) and 444 bi-allelic pollen (from 3 plants) were used for statistical analysis. (TIF) [file pgen.1006085.s008.tif]

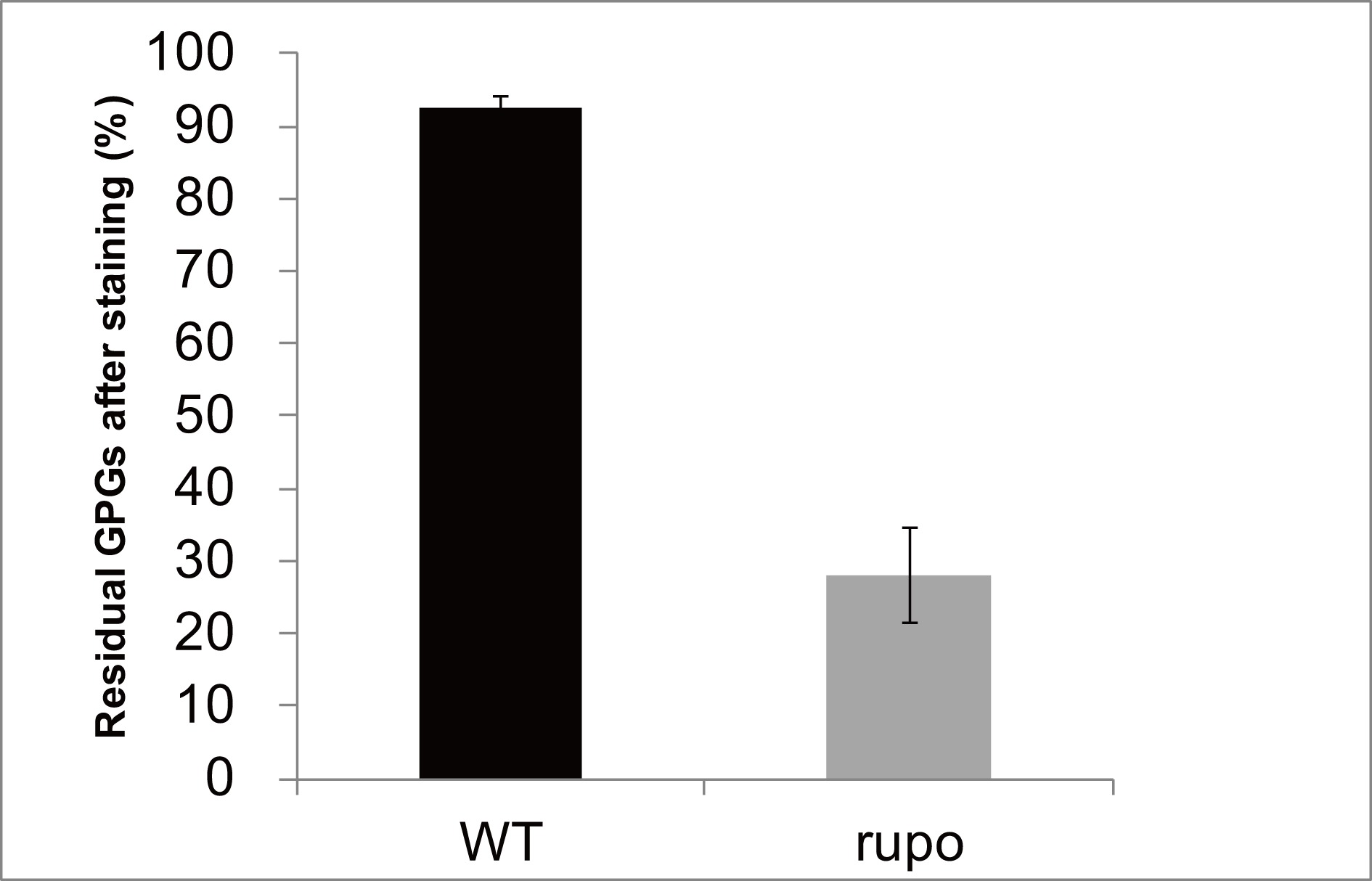

Supplement: S9 Fig — After aniline blue staining, residual germinated pollen grains (GPGs) and non-germinated pollen grains that attached to the stigma were counted separately. 544 wild-type pollen and 138 rupo pollen were used for statistical analysis. The results are presented as mean±s.e.. (TIF) [file pgen.1006085.s009.tif]

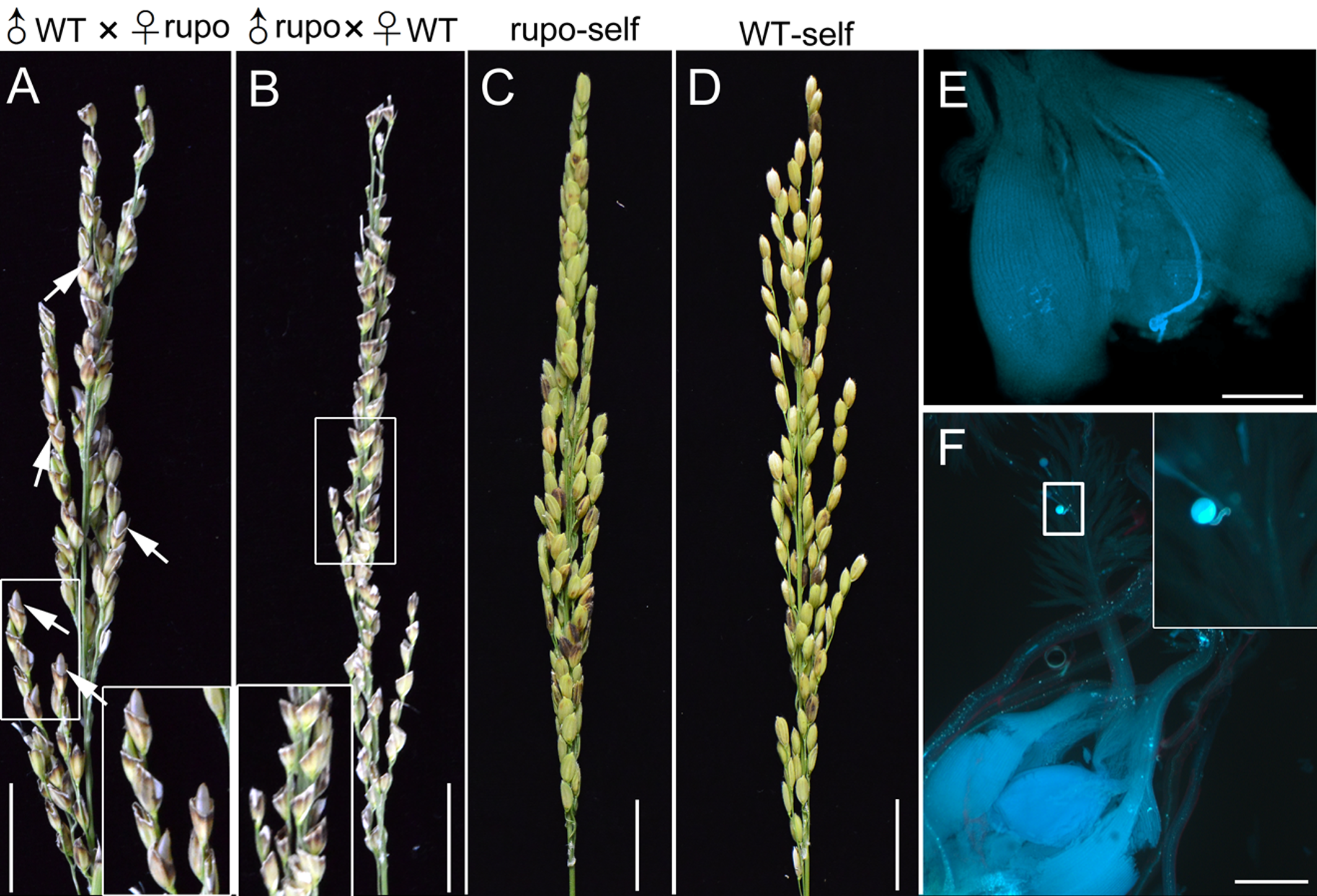

Supplement: S10 Fig — (A) Wild-type was used as pollen donor, bi-allelic as pollen receptor. White arrows indicate florets that produce seeds (B) Bi-allelic was used as pollen donor, wild-type as pollen receptor. Note that all glumes are empty. (C) Image of a bi-allelic spike 30 days after self-pollination. (D) Image of a wild-type spike 30 days after self-pollination. (E) Aniline blue staining shows a wild-type pollen tube targeting a bi-allelic micropyle. (F) A bi-allelic pollen grain germinated in a wild-type pistil but failed to reach the ovule. Scale bars, 2 cm in (A-D), 200 μm in (E,F). (TIF) [file pgen.1006085.s010.tif]

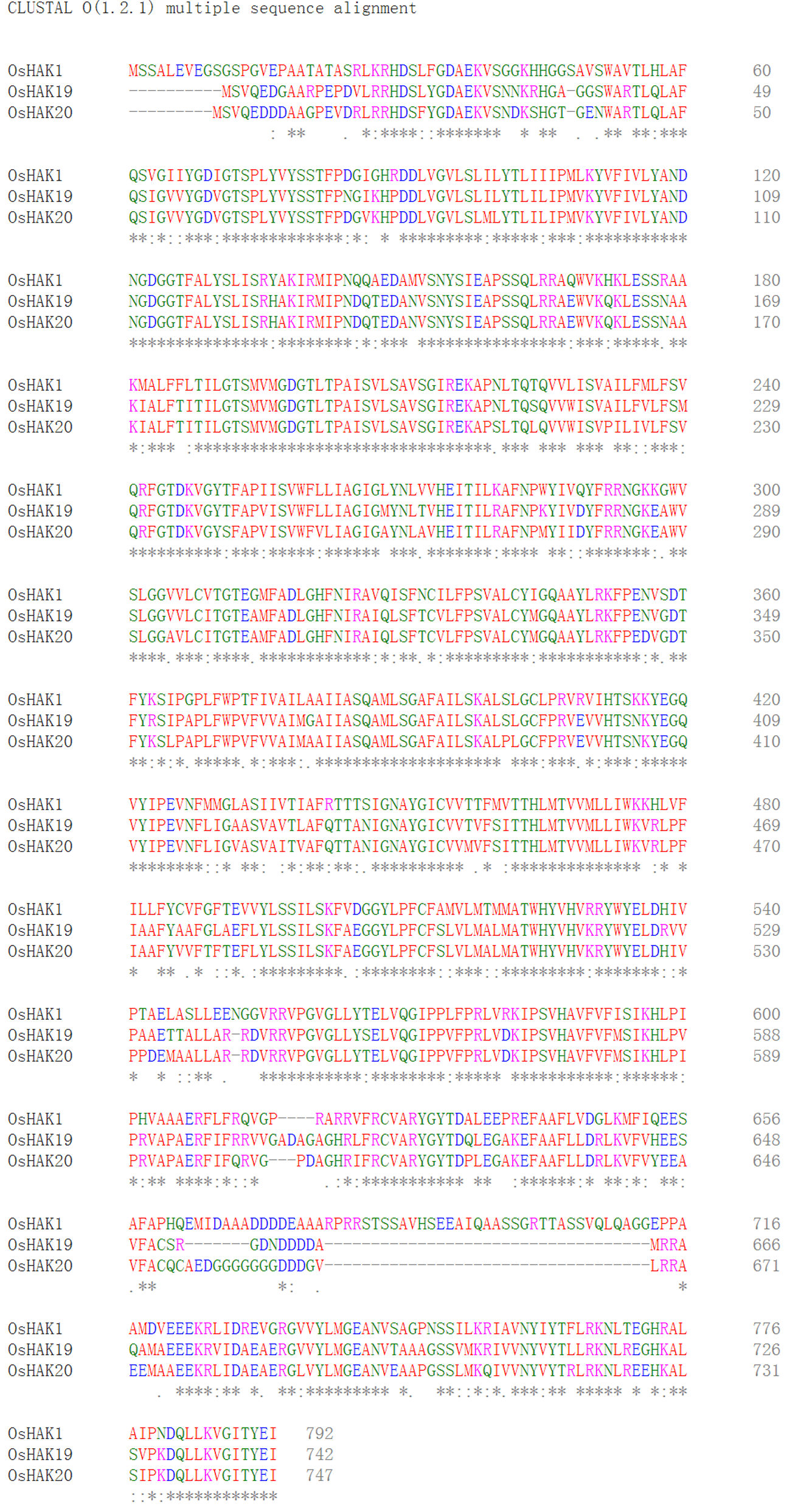

Supplement: S11 Fig — Protein sequence alignment of OsHAK1, OsHAK19 and OsHAK20 was performed with Clustal Omega program. Asterisks indicate identical amino acids. The dots indicate similar amino acids. Note that OsHAK1 contains an extra 35 amino acid residues (678-712aa) in the C-terminus. (TIF) [file pgen.1006085.s011.tif]

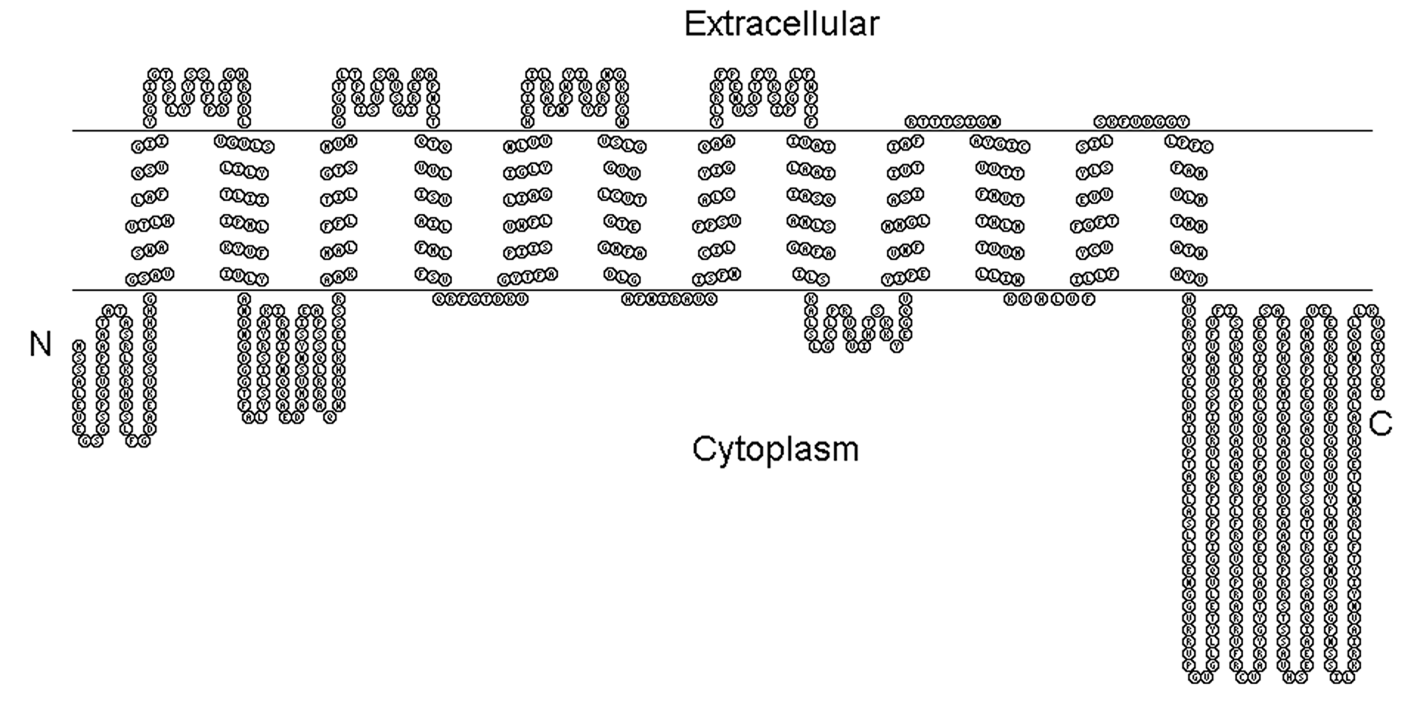

Supplement: S12 Fig — OsHAK1 protein contains 792 amino acid residues and 12 transmembrane domains, with N- and C-terminus inside the cytoplasm. Topology and transmembrane domains were predicted by using HMMTOP 2.1 software. (TIF) [file pgen.1006085.s012.tif]

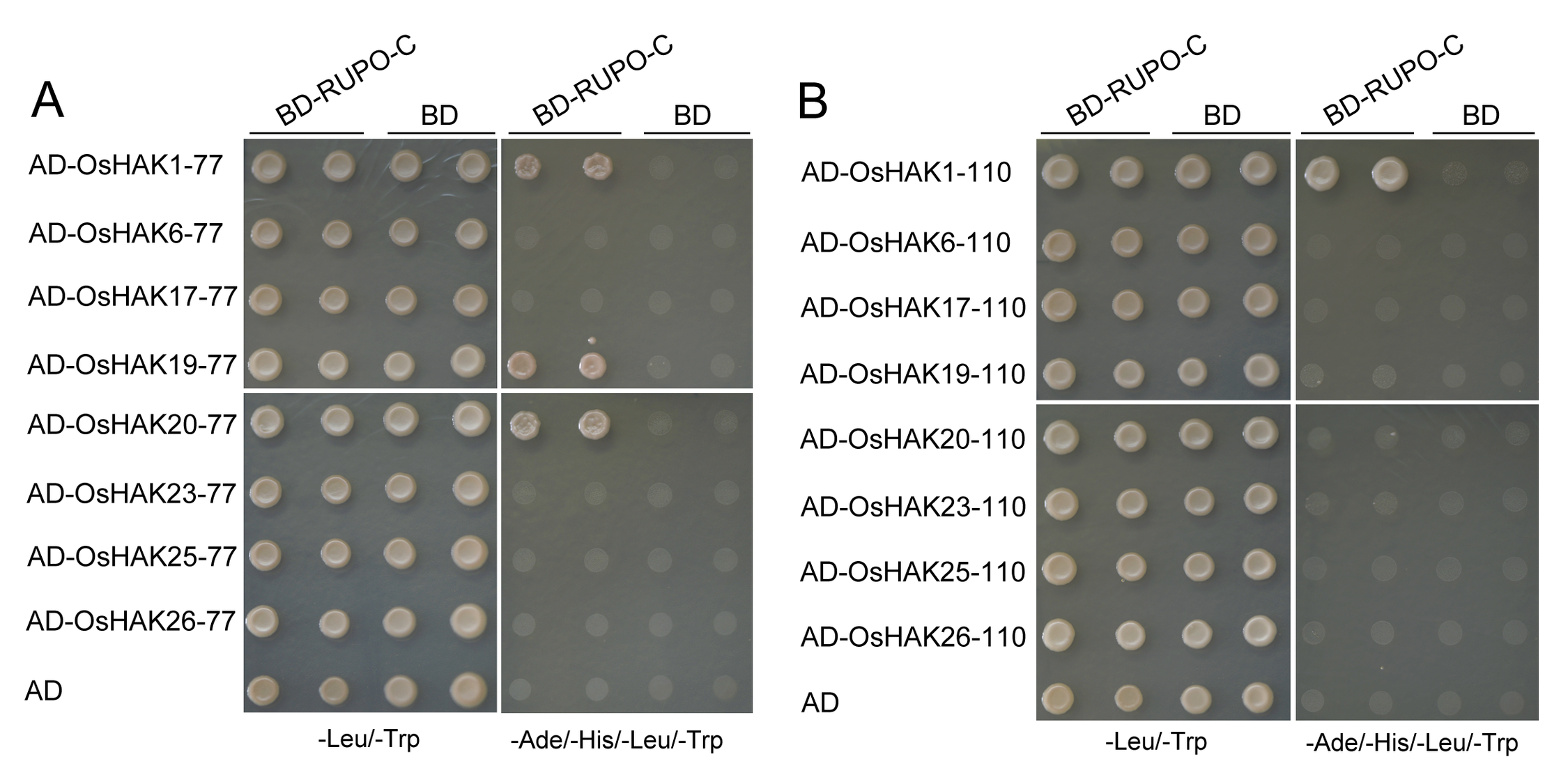

Supplement: S13 Fig — The C-tails of OsHAK1, OsHAK6, OsHAK17, OsHAK19, OsHAK20, OsHAK23, OsHAK25, and OsHAK26 were cloned from total RNA of mature pollen grains. The 77 aa length (A) and 110 aa length (B) C tails were used for the interaction assays. (TIF) [file pgen.1006085.s013.tif]

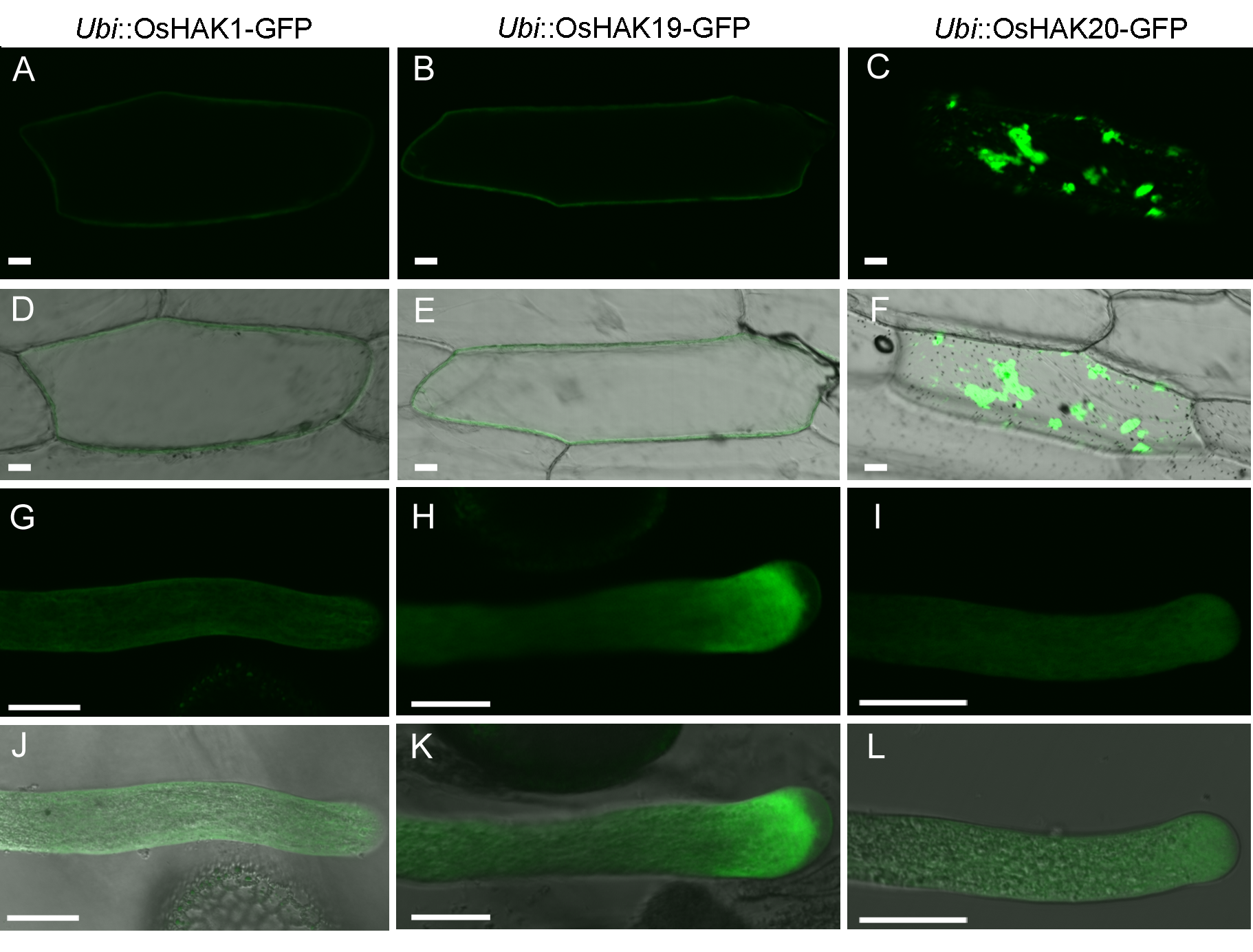

Supplement: S14 Fig — (A to F) Subcellular localization of the Ubi::OsHAKs-GFP fusion proteins in onion epidermal cells. (A) Single confocal section and (D) bright-field image of the cell bombarded with Ubi::OsHAK1-GFP plasmid. (B) Single confocal section and (E) bright-field image of the cell bombarded with Ubi::OsHAK19-GFP. (C) Single confocal section and (F) bright-field image of the cell transformed with Ubi::OsHAK20-GFP. (G to L) Transient expression of Ubi::OsHAK1-GFP (G,J), Ubi::OsHAK19-GFP (H,K), and Ubi::OsHAK20-GFP (I,L) in lily pollen tubes. Scale bars, 20 μm. (TIF) [file pgen.1006085.s014.tif]

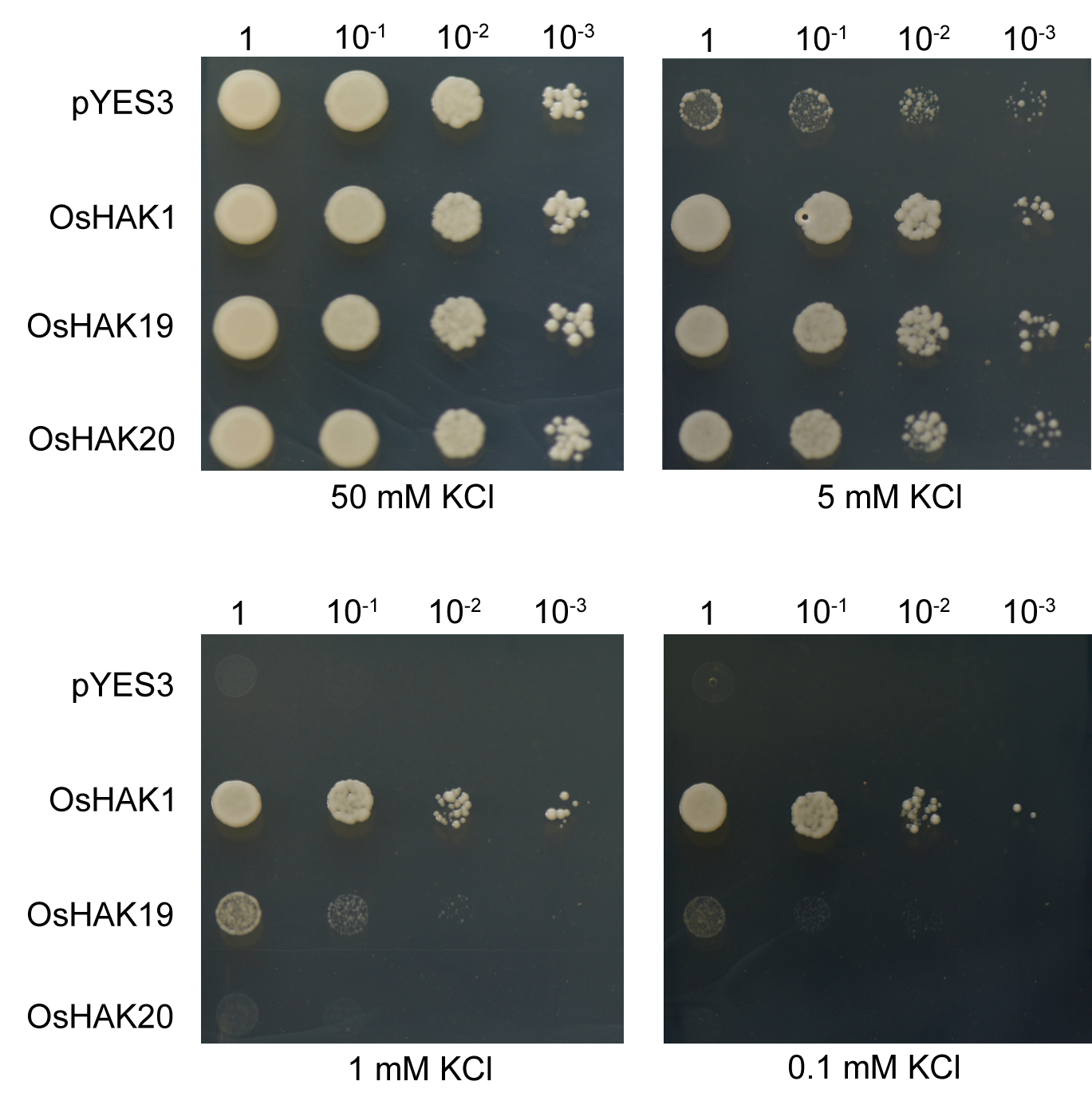

Supplement: S15 Fig — R5421 strain were transformed with OsHAK1/19/20 or expression vector pYES3 (control), and the transformants were dropped onto AP medium in 1:10 serial dilutions. (TIF) [file pgen.1006085.s015.tif]

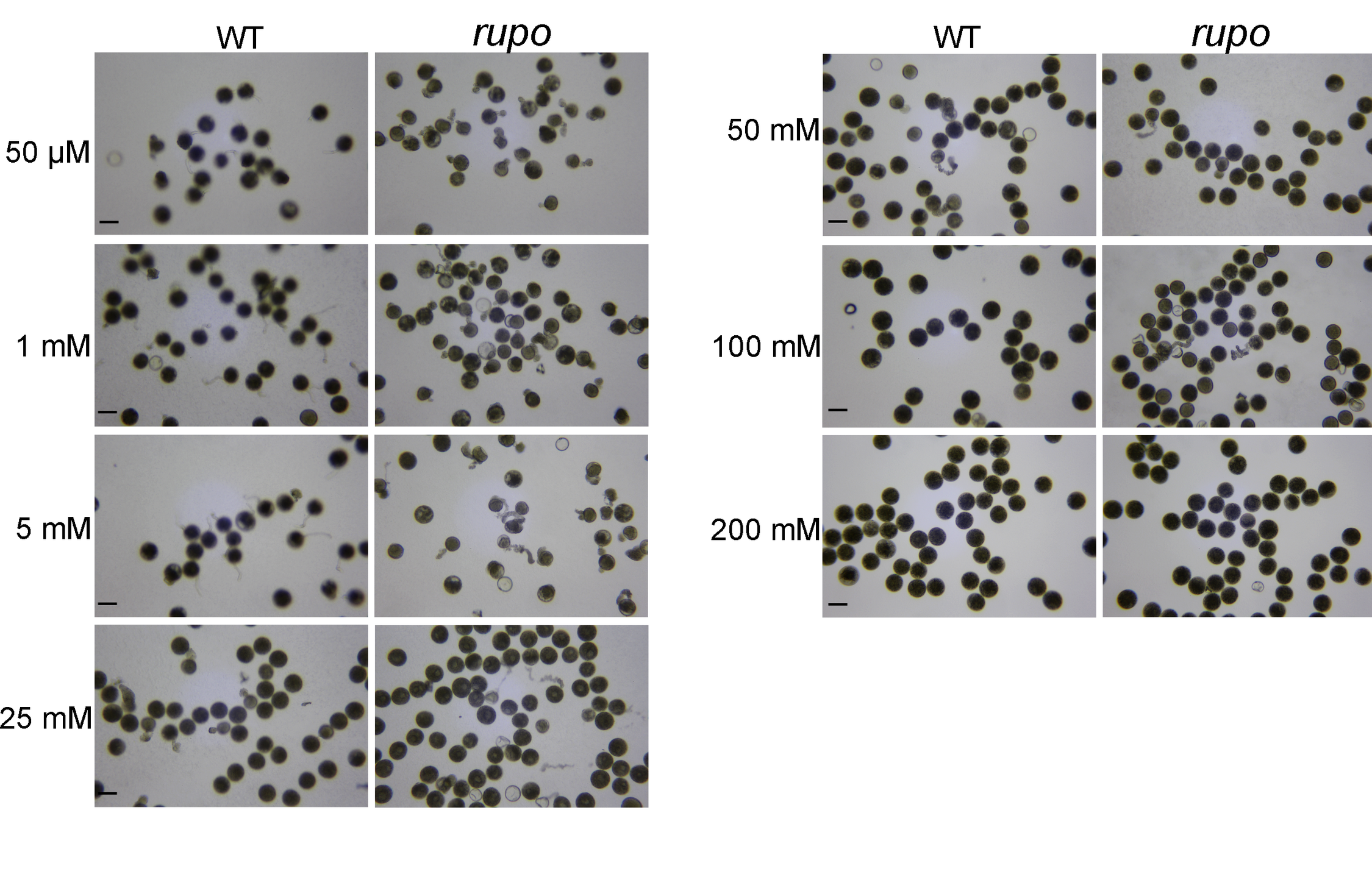

Supplement: S16 Fig — The original pollen germination medium contained 50 μm K+. The KCl concentrations are indicated. Scale bars, 50 μm. (TIF) [file pgen.1006085.s016.tif]
