# Supplementary material for: Receptor-Like Kinase RUPO Interacts with Potassium Transporters to Regulate Pollen Tube Growth and Integrity in Rice
Source: PLoS Genet. 2016 Jul 22;12(7):e1006085. doi: 10.1371/journal.pgen.1006085 (PMC4957769; doi:10.1371/journal.pgen.1006085)
Supplement: S1 Table — aThe amino acid sequence of phosphorylated peptides, where Ser/Thr phosphorylation and carbamidomethyl modification are denoted by [Pho],[CAM] respectively. bThe experimentally measured monoisotopic mass for the precursor ion fragmented. cThe difference in mass between the precursor MW and the theoretical MW of the matching peptide sequence. dPeptide confidence given by ProteinPilot 4.5. eMascot score. Individual ion score >34 (for trypsin) or score >29 (for Glu-C) means the probability of random match is less than 0.05. In total, the MS/MS data covered > 96.95% amino acid sequence of the intracellular domain, including all Ser/Thr/Tyr residues except for Tyr- 588. (PDF) [file pgen.1006085.s017.pdf]

Table S1. Identification of 10 *in vitro* phosphorylation sites of RUPO.

Peptide sequence[a]	Position	Prec MW[b]	ΔMass[c]	Confidence[d]	Score[e]
SGYTFS[Pho]STLGLGR	494 S	1424.6334	-0.0003	99	81
SGYTFSS[Pho]TLGLGR	495 S	1424.6387	0.0049	99	67
IQAAT[Pho]KNFE	512 T	1100.4880	-0.0022	99	33
IDDGT[Pho]KVAVKRGNPQSE	538 T	1892.9008	0.0016	99	71
DGPGMNQLHVS[Pho]TAVK	673 S	1632.7361	0.0029	99	76
DGPGMNQLHVST[Pho]AVK	674 T	1632.7354	0.0022	99	73
TLC[CAM]ARPPIDPQLPREQVS [Pho]LAE	726 S	2469.2075	-0.0009	99	80
LAGT[Pho]VNQESLNK	751 T	1352.6362	0.0025	99	88
LAGTVNQES[Pho]LNK	756 S	1352.6331	-0.0007	99	85
C[CAM]LAEFGS[Pho]DR	773 S	1133.4200	-0.0013	99	43

[a]The amino acid sequence of phosphorylated peptides, where Ser/Thr phosphorylation and carbamidomethyl modification are denoted by [Pho],[CAM] respectively. [b]The experimentally measured monoisotopic mass for the precursor ion fragmented. [c]The difference in mass between the precursor MW and the theoretical MW of the matching peptide sequence. [d]Peptide confidence given by ProteinPilot 4.5. [e]Mascot score. Individual ion score >34 (for trypsin) or score >29 (for Glu-C) means the probability of random match is less than 0.05. In total, the MS/MS data covered > 96.95% amino acid sequence of the intracellular domain, including all Ser/Thr/Tyr residues except for Tyr- 588.
