# Supplementary material for: Receptor-Like Kinase RUPO Interacts with Potassium Transporters to Regulate Pollen Tube Growth and Integrity in Rice
Source: PLoS Genet. 2016 Jul 22;12(7):e1006085. doi: 10.1371/journal.pgen.1006085 (PMC4957769; doi:10.1371/journal.pgen.1006085)
Supplement: S2 Table — (PDF) [file pgen.1006085.s018.pdf]

Table S2. List of primers.

Description	Name	Sequence
**Genotyping *rupo+/-***	12426F1	AGGTCCTTGCCGTAGATGTG
	12426R1	AGGTGGTGTACCGGATCAAC
	Ngus-RB	AACGCTGATCAATTCCACAG
**Genomic cloning**	NF4	TCACTATGTATAACTTAATGTTGCACCAACAGTCAAC
	NR4	CCCAACCCCTCCTCTCACAACCCAA
**Promoter**	LF1	TAGTTGGAATGGGTTCGAATCACTATGTATAACTTAATGTTGCACCAACA
	LR2	TTATGGAGTTGGGTTCGAAGGTGGTGGGGTGGCCTACG
**RT-PCR**	RLIP1-LP2	GAGCCCGAGCTTCAACATG
	RLIP1-RP	TCTCGGTGTTGAACTCGTTG
**qPCR**	RLIP1-q1F	TCGCCATCGCCAATCCAAGACA
	RLIP1-q1R	TCGATCGTCGCGGTGGAACG
	18SF	CGGCTACCACATCCAAGGAA
	18SR	TGTCACTACCTCCCCGTGTCA
**CRISPR lines**	127CF2	CACTTCGTCCGCCTCTACCTCTTC
	127CR2	GTTGATCCGGTACACCACCT
**Complementation**	F(HindIII)	AAGCTTGCACCAACAGTCAACCGAAACAT
	R（EcoRI）	GAATTCATCCCTGGTTACTTGATTAGTCTGTG
	127RP	TCTCGGTGTTGAACTCGTTG
	RB	AACGCTGATCAATTCCACAG
	d127LP	CGGCGTACTACAAGGACATCGTCGT
	d127RP-1	TAGCGTTGTGATGGAGTGCATTCCAG
	d127RP-2	AGGTTAACCGCGTTGCTAAACCAGC
**pET-RUPO-N**	127NF1	TTAGAATTCCAGGGGCGGCCGGTCACC
	127NR1	ACACTCGAGGCGGCTGCCGCTGCCGTC
**Y2H related**		
**Library construction**	CDSIII	ATTCTAGAGGCCGAGGCGGCCGACATG-d(T)30VN
	SMARTIII	AAGCAGTGGTATCAACGCAGAGTGGCCATTATGGCCGGG
**BD 515-845**	127YF2	ACTGAATTCTTCGAGGAGAGCGCCATC
	127YR2	CACGGATCCTCACCTCCCCTTCATGTC
**BD453-845**	127YF3	ACTGAATTCTGGTACAAGCGGCCCCAG
	127YR2	CACGGATCCTCACCTCCCCTTCATGTC
**BD515-820**	127YF2	ACTGAATTCTTCGAGGAGAGCGCCATC
	127YR3	CACGGATCCTCAGCCGGAGCCGGAG
**BD453-820**	127YF3	ACTGAATTCTGGTACAAGCGGCCCCAG
	127YR3	CACGGATCCTCAGCCGGAGCCGGAG
**BD453-708**	127YF3	ACTGAATTCTGGTACAAGCGGCCCCAG
	127YR4	ATAGGATCCTCACTCGAGGAGCACGACG
**BD515-708**	127YF2	ACTGAATTCTTCGAGGAGAGCGCCATC

	127YR4	ATAGGATCCTCACTCGAGGAGCACGACG
**BD453-788**	127YF3	ACTGAATTCTGGTACAAGCGGCCCCAG
	127YR5	ATAGGATCCTCACGCGTACTCGAGGTTC
**BD515-788**	127YF2	ACTGAATTCTTCGAGGAGAGCGCCATC
	127YR5	ATAGGATCCTCACGCGTACTCGAGGTTC
**AD-OsHAK1-C**	PotassYF2	ATAGAATTCACGAGCTCGGCCGTGC
	PotassYR1	CGCGGATCCCTAGATTTCATACGTGA
**AD-OsHAK19-C**	PTF4	ATAGAATTCGCCGCGTTCCTCCTCGA
	PTR2	CGCGGATCCCTAAATCTCATAGGTGATC
**AD-OsHAK20-C**	PTF5	ATAGAATTCCTCAAGGTGTTCGTCTAC
	PTR3	CGCGGATCCCTAAATCTCATAAGTGATC
**Kinase activity related**		
**MBP-RUPO515**	GSTKinaseF	TCTGGATCCTTCGAGGAGAGCGCCATC
	GSTKinaseR	TCTGAATTCCCTCCCCTTCATGTCGGC
**MBP-RUPO**	MBPF1	TCTGGATCCCAG GGG CGG CCG GTC ACC
	HiskinaseR	TCTAAGCTTCCTCCCCTTCATGTCGGC
**Point mutation**	K543R For	CCAAGGTCGCCGTGAGGCGCGGGAAC
	K543R Rev	CTCACGGCGACCTTGGTGCCGTCGTCG
**Localization**		
**35S::RUPOΔC-GFP**	127DWF1	ATACTCGAGATGCGCCGCCGCGGGAG
	127DWR1	CCGACTAGTCCGAGGCCGAGGGTGGAG
**Ubi::RUPO-GFP**	127DWF2	ATAGGATCCATGCGCCGCCGCGGGAG
	127DWR2	ATAACTAGTGGCTGCAGCGGCCCTCCCCTTCATGTCG
**Pull down related**		
**GST-RUPO-C**	GSTKinaseF	TCTGGATCCTTCGAGGAGAGCGCCATC
	HiskinaseR	TCTAAGCTTCCTCCCCTTCATGTCGGC
**MBP-OsHAK1-C**	MBPHAKF1	TATGGATCCACGAGCTCGGCCGTGC
	MBPHAKR1	GAGAAGCTTCTAGATTTCATACGTGA
**MBP-OsHAK19-C**	MBPHAKF2	TATGGATCCGCCGCGTTCCTCCTCGA
	MBPHAKR2	CGCAAGCTTCTAAATCTCATAGGTGATC
**MBP-OsHAK20-C**	MBPHAKF3	TATGGATCCCTCAAGGTGTTCGTCTAC
	MBPHAKR3	GGCAAGCTTCTAAATCTCATAAGTGATC
**Co-IP related**		
**FLAG-RUPO-C**	IPF2	ATAACTAGTAGATTACAAGGATGACGACGATAAGTCGAGGAGAGCGCCATC
	IPR1	ATCGGATCCTCACCTCCCCTTCATGTC
**GFP-OsHAK1-C**	IPKTF1	GACTCTAGACACGAGCTCGGCCGTGC
	IPKTR1	CGCGGATCCCTAGATTTCATACGTGA
**GFP-OsHAK19-C**	IPKTF2	GACTCTAGACGCCGCGTTCCTCCTCGA
	IPKTR2	CGCGGATCCCTAAATCTCATAGGTGA
**GFP-OsHAK20-C**	IPKTF3	GACTCTAGACCTCAAGGTGTTCGTCTAC
	IPKTR3	CGCGGATCCCTAAATCTCATAAGTGA
